# CircASH1L inhibits ferroptosis and enhances cisplatin resistance by sponging miR-515-5p to regulate cell cycle-related CDCA7/RRM2 in ovarian cancer cells

**DOI:** 10.3389/fphar.2025.1563869

**Published:** 2025-06-24

**Authors:** Lu Feng, Xinru Zou, Longyu Tang, Yijun Yuan, Tianwen He, Bin Su, Ying Tang, Jiang Wang, Kang Liu, Jun Li

**Affiliations:** ^1^ Department of Obstetrics and Gynecology, The Second Clinical Medical College of North Sichuan Medical College, Nanchong Central Hospital, Beijing Anzhen Nanchong Hospital of Capital Medical University, Nanchong, Sichuan, China; ^2^ The Second Clinical Medical College of North Sichuan Medical College, Institute of Tissue Engineering and Stem Cell Research, Nanchong Central Hospital, Beijing Anzhen Nanchong Hospital of Capital Medical University, Nanchong, Sichuan, China

**Keywords:** circASH1L, cisplatin resistance, ovarian cancer, ferroptosis, miR-515-5p, CDCA7, RRM2

## Abstract

**Background:**

Platinum chemotherapy, particularly cisplatin, has been the standard treatment for ovarian cancer. However, the development of resistance to cisplatin is a significant challenge during treatment. Circular RNAs (circRNAs) are a class of non-coding RNAs with a circular structure and have been implicated in regulating ferroptosis and chemoresistance. Despite the increasing recognition of circRNAs in cancer progression, the role of circASH1L in ferroptosis and cisplatin resistance in ovarian cancer remains poorly understood.

**Methods:**

RNA sequencing (RNA-seq) was utilized to identify differentially expressed circRNAs in ovarian cancer cells. Cell survival and invasion were assessed using CCK-8 and transwell assays, while apoptosis, cell cycle progression, and lipid peroxidation were analyzed by flow cytometry. Levels of GSH, MDA, and iron ions were measured using appropriate kits. qRT-PCR and Western blot analyses were performed to evaluate the expression of relevant RNAs and proteins. The clinical relevance of circASH1L/miR-515-5p/CDCA7 axis in ovarian cancer patients was analyzed using public datasets. Molecular interactions were confirmed through dual-luciferase reporter assays, RNA immunoprecipitation (RIP), and co-immunoprecipitation (Co-IP). *In vivo*, the effects of circASH1L on ferroptosis and chemoresistance were evaluated using a xenograft mouse model.

**Results:**

circASH1L expression was downregulated upon erastin treatment and significantly upregulated in cisplatin-resistant A2780/DDP and SKOV3/DDP cells. Silencing circASH1L reversed cisplatin resistance by reducing cell viability and invasion, while promoting apoptosis and ferroptosis. Mechanistically, circASH1L was found to act as a sponge for miR-515-5p, which in turn regulates the CDCA7/RRM2 axis. Rescue experiments demonstrated that inhibiting miR-515-5p or overexpressing CDCA7 blocked the effects of circASH1L silencing. Moreover, CDCA7 could interact with RRM2 and inhibit RRM2 degradation, which contributed to reducing cell cycle arrest and ferroptosis resistance. The clinical analysis showed circASH1L, and CDCA7/RRM2 expression was positive correlated with drug resistance and worse survival rate, while miR-515-5p expression was on the contrary. *In vivo*, silencing circASH1L enhanced cisplatin sensitivity by inducing ferroptosis.

**Conclusion:**

Our study demonstrates that silencing circASH1L alleviates cisplatin resistance in ovarian cancer cells. The underlying mechanism involves the upregulation of miR-515-5p, which targets the CDCA7/RRM2 axis, leading to cell cycle modulation and the induction of ferroptosis. Targeting the circASH1L/miR-515-5p/CDCA7 pathway offers new insights into the relationship between ferroptosis and chemoresistance, presenting a promising strategy to overcome chemoresistance in ovarian cancer.

## 1 Introduction

Ovarian cancer is a complex and highly fatal disease affecting women worldwide, characterized by poor prognosis and high mortality rates (Cai et al., 2023). Due to the lack of specific symptoms in its early stages, ovarian cancer is often diagnosed at advanced stages (III or IV), with a recurrence rate of approximately 70% (Sung et al., 2021; Ali et al., 2023). Currently, the standard treatment for newly diagnosed patients includes primary cytoreductive surgery followed by chemotherapy for maintenance (Abujamous et al., 2024). Cisplatin, a platinum-based chemotherapeutic agent, is widely used in the treatment of various cancers and is considered the first-line therapy for ovarian cancer (Jiang et al., 2024). However, chemotherapy frequently leads to the development of drug resistance, particularly in advanced-stage patients, significantly limiting its therapeutic efficacy (Cai et al., 2023). Therefore, developing new therapeutic strategies to overcome drug resistance and improve survival outcomes for ovarian cancer patients is of paramount importance.

Ferroptosis, a recently identified form of regulated cell death, is characterized by iron accumulation and lipid peroxidation (Dixon et al., 2012; Chen et al., 2021). Inducing ferroptosis in tumor cells has emerged as a promising anticancer strategy, and significant progress has been made in understanding its mechanisms. For example, the protein translation inhibitor thiostrepton has been shown to inhibit cell proliferation and clonogenesis in pancreatic cancer by inducing ferroptosis via the STAT3/GPX4 pathway (Zhang et al., 2022). Additionally, an oral peptide that attenuates PD-1/PD-L1 signaling has been reported to induce ferroptosis, showing great potential for cancer immunotherapy (Yang et al., 2024a). In ovarian cancer, elevated ROS levels have been observed in various cancer cell lines, suggesting that these cells may be particularly susceptible to ferroptosis (Wang X. et al., 2024). Our previous studies demonstrated that artesunate induces ferroptosis in ovarian cancer cells through the HOXC11/PROM2/PI3K/AKT pathway, offering a potential therapeutic avenue (Ding et al., 2023; Li et al., 2024). However, recent studies have shown that MEX3A, an RNA-binding protein, inhibits ferroptosis by promoting p53 degradation, thereby aggravating ovarian cancer progression (Wang and Chen, 2023).

Emerging evidence suggests that ferroptosis may play a significant role in overcoming cisplatin resistance. For instance, activating transcription factor 3 (ATF3) has been shown to promote ferroptosis and enhance cisplatin sensitivity in gastric cancer by inhibiting the Nrf2/Keap1/xCT pathway (Fu et al., 2021). Another study demonstrated that the LGR4 monoclonal antibody, which blocks the Wnt signaling pathway, activates ferroptosis in colorectal cancer, thus alleviating acquired cisplatin resistance (Zheng et al., 2024). Similarly, co-treatment with shikonin and cisplatin has been shown to activate HMOX1 (Heme oxygenase 1) and promote iron accumulation, inducing ferroptosis and mitigating cisplatin resistance in ovarian cancer (Ni et al., 2023). However, it has also been reported that blocking ferroptosis signaling may promote cell survival, peritoneal metastasis, and facilitate cisplatin resistance in ovarian cancer (Zhang and Li, 2023). These findings suggest that targeting ferroptosis could be a promising strategy to improve treatment outcomes and overcome drug resistance in ovarian cancer.

Circular RNAs (circRNAs) are a novel class of non-coding RNAs characterized by a stable circular structure, which plays critical roles in various biological processes, including the regulation of gene expression and cell death (Qu et al., 2015). Recent studies have highlighted the involvement of circRNAs in ferroptosis regulation. For example, circACAP2 regulates GPX4, a key protein in ferroptosis, through the miR-193a-5p/GPX4 axis, inhibiting ferroptosis and promoting the malignant progression of cervical cancer (Liu et al., 2022). In lung cancer, circP4HB promotes tumorigenesis by targeting the miR-1184/SLC7A11 axis to inhibit ferroptosis and enhance GSH synthesis (Pan et al., 2022). Additionally, circRNAs have been implicated in chemotherapy resistance by modulating ferroptosis. In gastric cancer cells, circHIPK3 contributes to cisplatin resistance by inhibiting autophagy-dependent ferroptosis, while silencing circHIPK3 enhances ferroptosis through the miR-508-3p/Bcl-2/Beclin1/SLC7A11 axis, reducing cisplatin resistance (Shang et al., 2023). In ovarian cancer, circSnx12 inhibits ferroptosis and renders ovarian cancer cells resistant to cisplatin treatment via the miR-194-5p/SLC7A11 axis (Qin et al., 2023). These findings suggest that targeting circRNAs may offer a powerful strategy to regulate the ferroptosis pathway, thereby enhancing the sensitivity of ovarian cancer patients to cisplatin therapy.

Although the role of circASH1L in inhibiting ferroptosis and promoting skin wound healing has been previously reported (Zha et al., 2023; Wang Q. et al., 2024), its function in tumors, particularly ovarian cancer, remains poorly understood. In this study, we observed that circASH1L was significantly downregulated in ovarian cancer cells treated with erastin, a known ferroptosis inducer. Interestingly, we also found that circASH1L was significantly upregulated in cisplatin-resistant ovarian cancer cell lines. These results suggest that circASH1L may play a critical role in regulating ferroptosis-related cisplatin resistance. Further investigation revealed that circASH1L inhibits ferroptosis and cell cycle arrest, thereby enhancing cisplatin resistance through the miR-515-5p/CDCA7/RRM2 axis. These findings not only deepen our understanding of circASH1L’s role in ovarian cancer but also offer novel insights into potential therapeutic strategies for overcoming cisplatin resistance.

## 2 Materials and methods

### 2.1 Cell culture and transfection

The human ovarian cancer SKOV3 cell line was purchased from the Cell Bank of the Chinese Academy of Sciences (Shanghai, China) and cultured in McCoy’s 5A medium (Gibco, 16600082), supplemented with 10% fetal bovine serum (FBS, Gibco, 10091155) and 1% penicillin-streptomycin (P/S) 100× Solution (HyClone, SV30010). The A2780 cell line was obtained from Guangzhou Jennio Biotech Co., Ltd. and maintained in RPMI-1640 medium (Wisent, 350-000-CL) supplemented with 10% FBS (Gibco, 10091155) and 1% P/S (HyClone, SV30010). All cells were cultured at 37°C in a humidified incubator with 5% CO_2_. To induce cisplatin (DDP) resistance, cells were treated with increasing concentrations of cisplatin (MCE, HY-17394). Cell transfections were performed using Lipofectamine^®^ RNAiMAX Reagent (Invitrogen, 13778-150) or Lipofectamine^®^ 2000 Reagent (Invitrogen, 11668-019) according to the manufacturer’s instructions.

### 2.2 RNA sequencing (RNA-seq) and data analysis

A2780 cells were treated with DMSO (control) or 5 µM erastin for 24 h (MCE, HY-15763). Total RNA was extracted using TRIzol reagent (Invitrogen, 15596026). RNA-seq libraries were constructed using the Collibri Stranded RNA Library Prep Kit (Invitrogen, A39117024) according to the manufacturer’s instructions. The libraries were then sequenced on an Illumina HiSeq 4,000 platform (Illumina, USA). Back-splicing reads were identified using both the find_circ and CIRCexplorer tools. The final circRNA dataset was generated by taking the intersection of the circRNA predictions from both tools. Differentially expressed genes (DEGs) between the control and erastin-treated groups were identified using the DESeq2 R package. Statistical significance for DEGs was determined with a |log_2_FC| > 1 and a *p*-value <0.01.

### 2.3 Quantitative real-time polymerase chain reaction (qRT‒PCR) analysis

Total RNA was extracted from cells using TRIzol reagent (Invitrogen, 15596026). For mRNA expression analysis, RNA was reverse-transcribed to cDNA using the PrimeScript™ RT Reagent Kit (Takara, RR037A) according to the manufacturer’s instructions. For miRNA expression analysis, cDNA was synthesized using the miRNA 1st Strand cDNA Synthesis Kit (Vazyme, MR101-02). qRT-PCR was performed on a QuantStudio 12K (Thermo Fisher Scientific, USA) using the TB Green^®^ Premix Ex Taq™ II FAST qPCR kit (Takara, CN830A). The qRT-PCR protocol included the following steps: denaturation at 95°C for 30 s, followed by 40 cycles of 95°C for 5 s, 60°C for 10 s, and a dissociation stage to generate a melt curve. GAPDH or U6 served as the internal control, and data were analyzed using the 2^−ΔΔCT^ method. RNase R (Ribonuclease R) treatment was conducted to validate circRNA circularization. Total RNA (5 μg) was incubated with or without 4 U of RNase R (Beyotime, R7092S) for 20 min at 37°C, followed by heat inactivation at 70°C for 10 min. The product was then analyzed by qRT-PCR. The primers used for qRT-PCR are listed in Supplementary File 1.

### 2.4 Fluorescence *in situ* hybridization (FISH)

The FAM-labeled circASH1L-specific probe and Cy3-labeled miR-515-5p-specific probe were obtained from GenePharma (Shanghai, China). For the FISH assay, cells were fixed with 4% paraformaldehyde, permeabilized with 0.5% Triton X-100, and incubated with the indicated probes at 37°C overnight in the dark. After washing, the cells were stained with DAPI to visualize the nuclei. Images were captured using a confocal microscope (Olympus, Japan). The sequences of the circASH1L and miR-515-5p probes are provided in Supplementary File 1.

### 2.5 Cell viability assay

Cells were seeded and cultured in 96-well plates overnight. Then, the cells were treated with a series of concentrations of cisplatin for 48 h. For the cell viability assay, 10 μL of Cell Counting Kit-8 (CCK-8) reagent (Vazyme, A311-01) was added to each well, and the cells were incubated for 3 h. Absorbance at 450 nm was measured using a microplate reader (Thermo Fisher Scientific, USA).

### 2.6 Flow cytometry analysis

Cell apoptosis was assessed using an Annexin V-FITC/PI Apoptosis Detection Kit (Vazyme, A211-02). Cells were collected and resuspended in 1× binding buffer. 5 μL of Annexin V-FITC and 5 µL of PI solution were added. After 10 min incubation, apoptosis was detected by flow cytometry (Agilent, USA). Lipid peroxidation levels were measured using BODIPY 581/591 C11 reagent (Invitrogen, D3861). Cells were collected and 5 µM BODIPY was added for 30 min of incubation in a cell incubator. Lipid peroxides were then detected by flow cytometry (Agilent, USA). For the cell cycle assay, cells were harvested, fixed with 70% cold ethanol overnight, washed, and incubated with a cell cycle staining solution. Cell cycle analysis was performed by flow cytometry (Agilent, USA).

### 2.7 Transwell assay

Cell invasion was assessed using a transwell chamber (Corning, 3,422) coated with Matrigel (BD Biosciences, 356234). Cells were collected and suspended in serum-free medium at a density of 2 × 10^5^/mL. A 200 µL aliquot of the cell suspension was added to the upper chamber of the Transwell, and complete medium (10% FBS) was added to the lower chamber. The cells were cultured for 48 h. Afterward, invasive cells were fixed with 4% paraformaldehyde and stained with crystal violet. Images (magnification 200×) were captured using a microscope (Olympus, Japan), and the number of invasive cells was quantified using ImageJ software.

### 2.8 Western blot analysis

Protein was extracted from cells or tissues using RIPA reagent (Beyotime, P0013) containing 1 mM PMSF (Beyotime, ST506). Protein concentration was determined using a BCA assay kit (Beyotime, P0012). 30 μg of protein were loaded onto an SDS-PAGE gel, and the proteins were subsequently transferred to a nitrocellulose membrane. The membranes were blocked with 5% nonfat milk and incubated with diluted primary antibodies at 4°C overnight. The dilution factors for the primary antibodies were as follows: GAPDH (Proteintech, 10494-1-AP) at 1:10,000; GPX4 (Proteintech, 67763-1-Ig) and RRM2 (Proteintech, 67006-1-Ig) at 1:5,000; SLC7A11 (Abcam, ab307601) at 1:1,000; and CDCA7 (Proteintech, 15249-1-AP) at 1:2,000. The dilution factor for the secondary antibodies (goat anti-mouse and goat anti-rabbit) was 1:5,000. Protein bands were detected using Pierce™ ECL Western blotting Substrate (Thermo Scientific, 32209), and images were acquired with a chemiluminescence imaging system (Bio-Rad, USA). Relative protein expression was quantified using ImageJ software based on the gray intensity values, with GAPDH as the internal control.

### 2.9 Phen Green™ SK (PGSK), malondialdehyde (MDA) and glutathione (GSH) detection

Intracellular iron ion (Fe^2+^) levels were assessed using the Phen Green™ SK (PGSK) reagent (Invitrogen, P14313). PGSK fluorescence is quenched upon binding to iron ions. Cells were washed with PBS and incubated with 10 µM PGSK for 20 min at 37°C. After washing with PBS, images (magnification 100×) were captured using a fluorescence microscope (Olympus, Japan). GSH and MDA levels were measured using a GSH assay kit (Nanjing Jiancheng Bioengineering Institute, A006-2-1) and an MDA assay kit (Beyotime, S0131S), following the manufacturer’s instructions.

### 2.10 Dual-luciferase reporter assay

For testing the interaction between circASH1L and miR-155-5p, the wild-type (circASH1L-WT) or mutant (circASH1L-MUT) sequences were cloned into the pmirGLO vector. For the miR-155-5p binding assay to the 3′UTR of CDCA7, the wild-type (CDCA7-WT) or mutant (CDCA7-MUT) sequences were cloned into the pmirGLO vector. SKOV3/DDP and A2780/DDP cells (1 × 10^5^ cells) were seeded in 24-well plates and cultured overnight. The WT or MUT vector (0.5 µg) was co-transfected with 100 nM of either negative control mimics or miR-155-5p mimics (GenePharma, Shanghai, China). After 48 h, luciferase activity was measured using the Dual-Luciferase Reporter Assay System (Promega, E1910) according to the manufacturer’s instructions.

### 2.11 RNA immunoprecipitation (RIP)

Cells were harvested and lysed with RIPA lysis buffer (Beyotime, P0013). Protein A + G beads (Beyotime, P2012) suspended in RIP wash buffer were incubated with Ago2 antibody (Proteintech, 67934-1-Ig) or normal IgG, followed by incubation with the cell extracts at 4°C overnight. The RNA-protein complexes were then eluted by treatment with proteinase K. RNA was extracted from the eluate using phenol-chloroform-isoamyl alcohol and precipitated by ethanol. The isolated RNA was reverse transcribed to cDNA and subjected to qRT-PCR. The data were normalized to the input group.

### 2.12 Co-immunoprecipitation (co-IP)

Cells were lysed with Western blotting and IP lysis buffer (Beyotime, P0013) containing protease inhibitor cocktails. Protein A + G beads (Beyotime, P2012) were incubated with RRM2 antibody (Proteintech, 67006-1-Ig) or Normal IgG. After protein concentration determination, the lysates were incubated with the bead-antibody complex. After overnight incubation, the immunoprecipitates were washed four times and boiled with 2× loading buffer. The protein levels of RRM2 and CDCA7 were analyzed by Western blot.

### 2.13 Differential expression analysis of cisplatin resistance-associated genes in ovarian cancer

To investigate genes associated with cisplatin resistance in ovarian cancer, transcriptome data and corresponding clinical information were obtained from the TCGA and GEO databases. The expression data were processed into transcripts per million (TPM) format and normalized using log_2_ (TPM+1). Based on the pharmacogenomic information from the Genomics of Drug Sensitivity in Cancer (GDSC) database, the chemotherapeutic response of each sample was estimated. Cisplatin sensitivity, indicated by half-maximal inhibitory concentration (IC50), was predicted using the pRRophetic R package (v4.2.2) with default ridge regression parameters, batch correction by Combat, and tissue type set as “all.” For genes with multiple probes, expression values were averaged.

Samples were divided into cisplatin-sensitive and cisplatin-resistant groups based on the predicted IC50 values, and differential expression analysis was conducted between the two groups. Statistical significance was assessed using the Wilcoxon rank-sum test, with *p* < 0.05 considered significant.

### 2.14 Survival analysis of key genes associated with prognosis in ovarian cancer

Survival analysis was performed using transcriptomic data and matched clinical information from ovarian cancer patients in the TCGA and GEO databases. Only samples with both gene expression and survival outcome data were retained, resulting in a final dataset of 300 patients for analysis.

Kaplan–Meier survival curves were generated using the survival package in R software (v4.2.2), with patient groups stratified based on gene expression levels. The log-rank test was applied to assess differences in overall survival between groups. In addition, univariate Cox proportional hazards regression was used to calculate the hazard ratio (HR) and 95% confidence interval (CI), quantifying the prognostic relevance of gene expression. A *p* value <0.05 was considered statistically significant.

### 2.15 Xenograft mouse model

Four-week-old female BALB/c nude mice were purchased from Vital River (Beijing, China). Stably downregulated circASH1L (sh-circASH1L) or negative control (sh-NC) A2780/DDP cells were established using lentivirus (GenePharma, Shanghai, China). After acclimatization, a total of 5 × 10^6^ sh-circASH1L or sh-NC A2780/DDP cells were subcutaneously injected into the right flank of the mice to establish the xenograft model. When tumors reached approximately 100 mm^3^, the mice were randomly assigned into four groups (n = 6 for each group): sh-NC + PBS, sh-circASH1L + PBS, sh-NC + cisplatin, and sh-circASH1L + cisplatin. Mice were intraperitoneally injected with PBS or 6 mg/kg cisplatin twice a week. Tumor length, width, and body weight were measured every 3 days. Tumor volume was calculated using the following formula: volume (mm^3^) = 1/2 × (length × width^2^). At the treatment endpoint, the mice were euthanized, and tumor tissues were collected for qRT-PCR, immunohistochemistry (IHC), Western blot, MDA, and GSH detection. IHC was performed as previously described using antibodies against GPX4, SLC7A11, CDCA7, and RRM2 (Li et al., 2024). All animal experiments were conducted in compliance with institutional guidelines and were approved by the Ethics Committee of North Sichuan Medical College (NSMC 2024 (118)).

### 2.16 Statistical analysis

Data are presented as the mean ± standard deviation (SD). All experiments were performed with at least three biological replicates. Graphing and statistical analysis were performed using GraphPad Prism 8.0.2 software. An unpaired t-test was used for comparisons between two groups. One-way ANOVA was used for comparisons between more than two groups. A *p*-value of <0.05 was considered statistically significant, with *p < 0.05. **p < 0.01, ***p < 0.001 indicating significant differences.

Statistical methods for bioinformatics analyses, including the Wilcoxon rank-sum test, log-rank test, Cox regression, are described in Sections 2.13 and 2.14.

## 3 Results

### 3.1 Identification and characterization of circASH1L as a potential ferroptosis-related factor in cisplatin-resistant ovarian cancer cells

**FIGURE 1:**
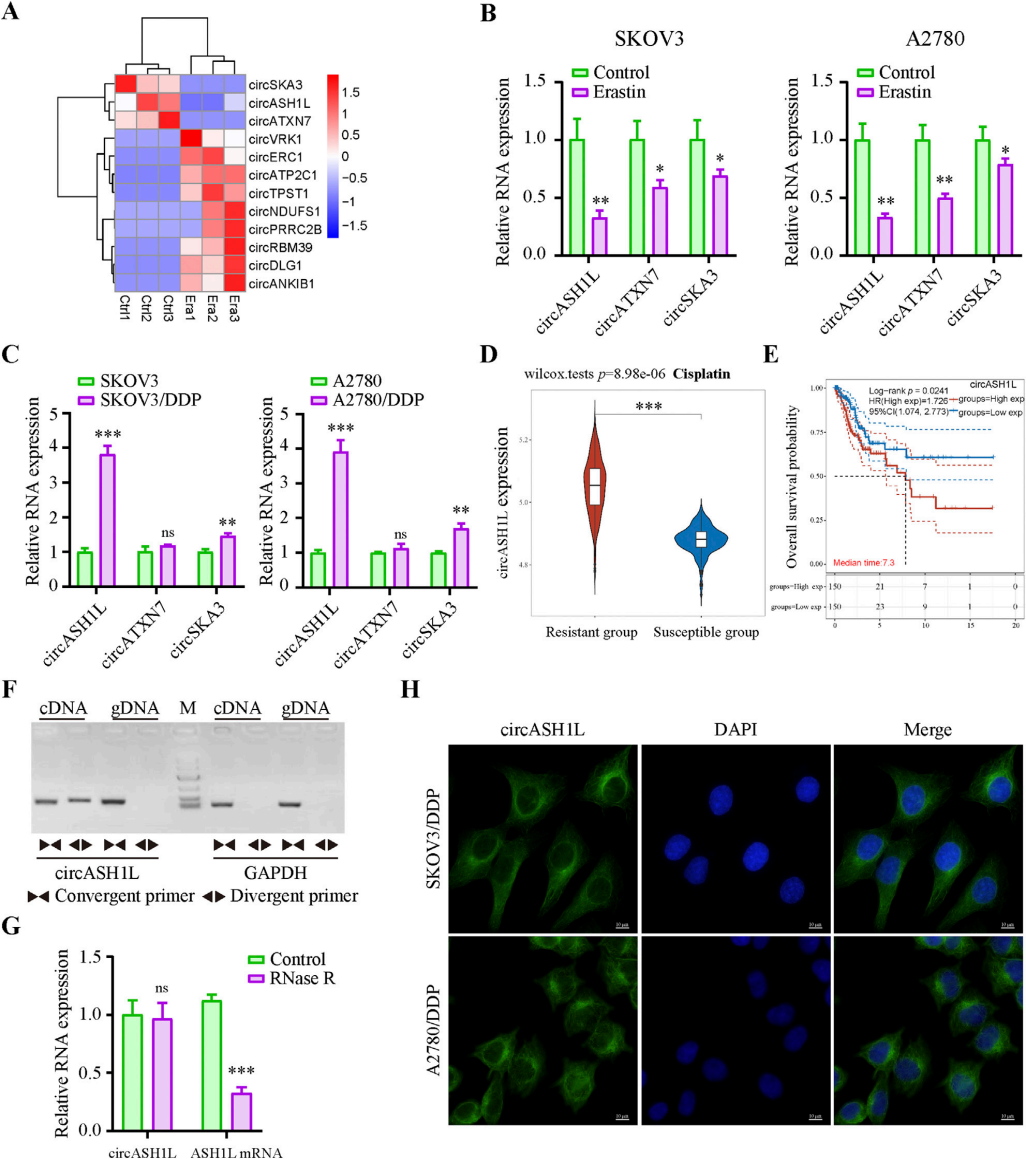
CircASH1L identified as a potential ferroptosis-related factor and upregulated in cisplatin-resistant ovarian cancer cells **(A)** Heatmap showed the differential expression of circRNAs (|log_2_FC| > 1, *p*-value <0.01). Era1-Era3 represented erastin-treated A2780 cells, and Ctrl1-Ctrl3 represented control A2780 cells. **(B)** qRT-PCR analysis of circASH1L, circATXN7 and circSKA3 expression in SKOV3 and A2780 cells treated with DMSO or 5 μM erastin for 24 h **(C)** qRT-PCR analysis of circASH1L expression in SKOV3, SKOV3/DDP, A2780 and A2780/DDP cells. **(D)** Box plot analysis of circASH1L expression in ovarian cancer patients stratified by cisplatin resistance status, based on TCGA and GEO datasets. **(E)** Kaplan–Meier survival curves depicting overall survival of ovarian cancer patients according to circASH1L expression levels. **(F)** PCR analysis of the back-spliced and linear forms of ASH1L expression in A2780 cells using convergent and divergent primers. **(G)** qRT-PCR analysis of circASH1L and linear ASH1L mRNA expression in A2780 cells with or without RNase R digestion. **(H)** Fluorescence *in situ* hybridization (FISH) experiments showed the cytoplasmic localization of circASH1L in SKOV3/DDP and A2780/DDP cells using antisense probe tagged with FAM. Scale bars = 10 µm. Data were analyzed as the mean ± SD of three independent experiments. *p < 0.05. **p < 0.01, ***p < 0.001.

To identify key circRNAs involved in the regulation of ferroptosis, RNA-seq was performed on ovarian cancer cell line A2780 treated with erastin. Three circRNAs, circASH1L (hsa_circ_0014592), circATXN7 (hsa_circ_0007761) and circSKA3 (hsa_circ_0000467), were significantly downregulated in the erastin-treated group compared to the control (Figure 1A). These findings suggest that these circRNAs may be involved in inhibiting ferroptosis. To further validate these results, qRT-PCR was performed to assess the expression of these circRNAs in A2780 and SKOV3 cells treated with DMSO or erastin. The results confirmed that the expression of three circRNAs were consistent with the RNA-seq data (Figure 1B). Since ferroptosis is closely linked to drug resistance, we next investigated the expression of these circRNAs in cisplatin-resistant ovarian cancer cells. A2780/DDP and SKOV3/DDP cells were established by sequential exposure to increasing concentrations of cisplatin, and their cisplatin sensitivity and IC50 values were determined after treatment with increasing concentrations of cisplatin (Supplementary Figure S1A). Notably, circASH1L was upregulated approximately 4-fold in both A2780/DDP and SKOV3/DDP cells, while the expression of circATXN7 and circSKA3 showed only slight increases (Figure 1C). To explore the clinical relevance of circASH1L, we performed bioinformatic analysis using ovarian cancer transcriptomic data and clinical annotations from the TCGA and GEO databases. Box plot analysis revealed that the expression of circASH1L was significantly higher in cisplatin-resistant group compared to susceptible group (*p* < 0.05) (Figure 1D). Furthermore, Kaplan–Meier survival analysis demonstrated that high circASH1L expression was significantly associated with shorter overall survival (*p* < 0.05) (Figure 1E). These findings suggest that circASH1L is closely associated with cisplatin resistance and may serve as a prognostic indicator in ovarian cancer.

The backsplicing site of circASH1L was identified to be formed from exon 3 of the ASH1L mRNA on chromosome 1q22 (Supplementary Figure S1C). The backsplice junction was further confirmed by Sanger sequencing (Supplementary Figure S1D). PCR analysis revealed that both divergent and convergent primers could amplify ASH1L fragments in cDNA, but only convergent primers could amplify the corresponding fragments in genomic DNA (gDNA) (Figure 1F). Additionally, qRT-PCR analysis demonstrated that circASH1L was resistant to RNase R digestion, while linear ASH1L mRNA was efficiently degraded by RNase R, further confirming the circular nature of circASH1L (Figure 1G). Furthermore, FISH experiments were performed to determine the subcellular localization of circASH1L. The results showed that circASH1L was predominantly localized in the cytoplasm of both A2780/DDP and SKOV3/DDP cells (Figure 1H). These findings suggest that circASH1L may play a role in regulating ferroptosis and cisplatin resistance in the cytoplasm of these cells.

### 3.2 Silencing circASH1L increased cisplatin sensitivity in ovarian cancer cells

**FIGURE 2:**
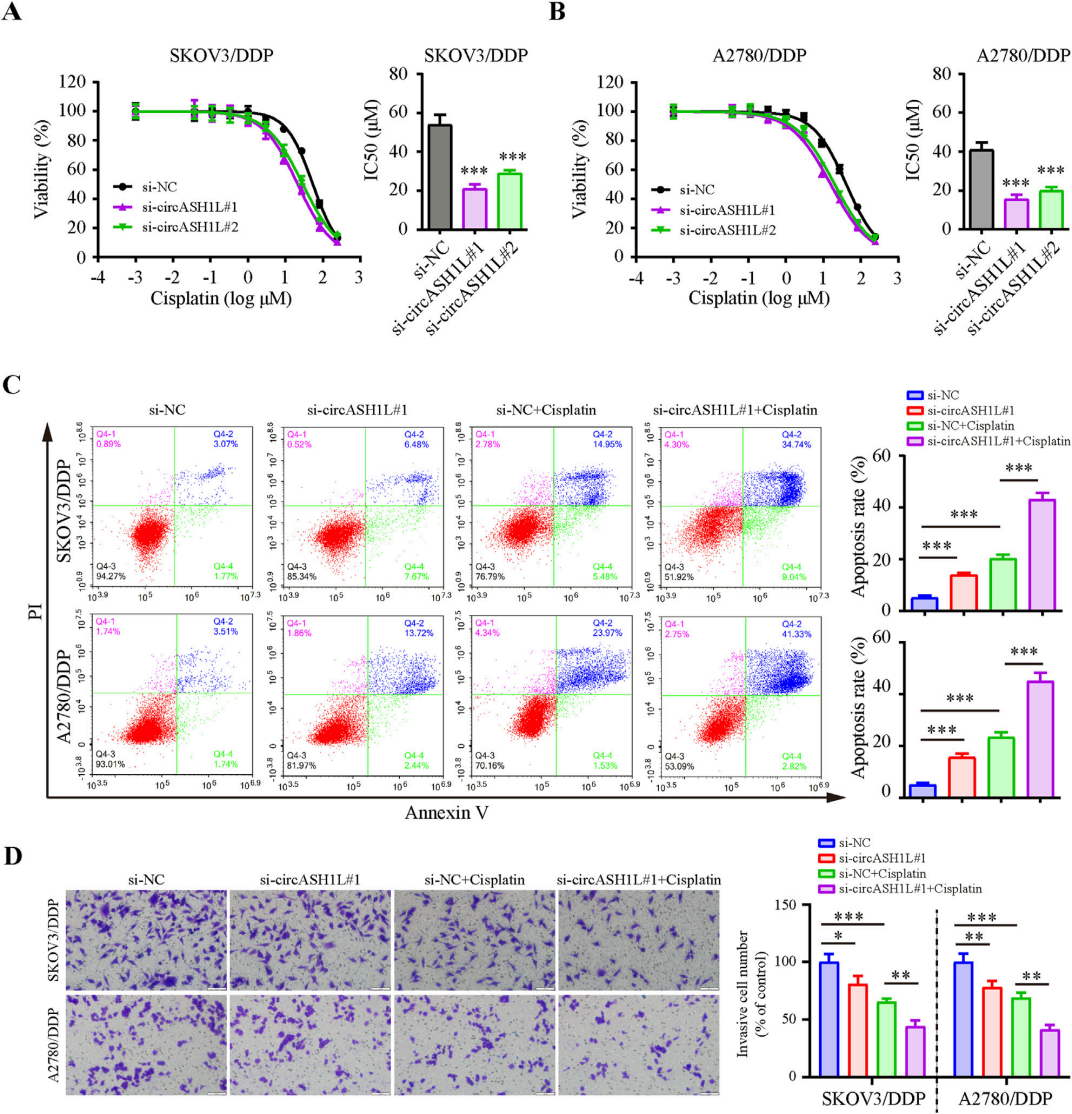
CircASH1L silencing inhibited proliferation and invasion, but promoted apoptosis in cisplatin-resistant ovarian cancer cells Cell viability (% of control) was assessed by CCK-8 assay following circASH1L knockdown with two different siRNAs in **(A)** SKOV3/DDP and **(B)** A2780/DDP cells, treated with various concentrations of cisplatin. The corresponding IC50 bar graph showed a significant reduction in cell viability and lower IC50 values for cisplatin in the circASH1L knockdown groups. **(C and D)** SKOV3/DDP and A2780/DDP cells were treated with either si-NC or si-circASH1L, with or without cisplatin. **(C)** Cell apoptosis was analyzed by flow cytometry using Annexin V/PI staining. **(D)** Transwell invasion assay was performed to assess cell invasion. Scale bars = 100 µm. Data were analyzed as the mean ± SD of three independent experiments. *p < 0.05. **p < 0.01, ***p < 0.001.

To investigate the role of circASH1L in cisplatin resistance, we silenced its expression in cisplatin-resistant ovarian cancer cells. qRT-PCR analysis demonstrated that all of three siRNAs effectively decreased circASH1L expression, with si-circASH1L#1 and si-circASH1L#2 showing superior silencing efficacy compared to si-circASH1L#3 (Supplementary Figure S2A). To assess whether circASH1L contributes to cisplatin resistance, A2780/DDP and SKOV3/DDP cells were treated with various concentrations of cisplatin following siRNA transfection. Notably, the IC50 values for cisplatin in the si-circASH1L groups were significantly lower compared to the NC siRNA group (Figures 2A,B). Based on these results, si-circASH1L#1 was selected for subsequent experiments. For the following functional assays, 15 μM cisplatin was used, which corresponds to approximately 20%–30% growth inhibition (IC20–30) in these cells. Moreover, silencing circASH1L resulted in increased cell apoptosis and reduced cell invasion compared to the NC siRNA transfection group. Importantly, silencing circASH1L further enhanced the effect of cisplatin, as the si-circASH1L#1 + cisplatin group exhibited increased apoptosis and decreased invasion compared to the si-NC + cisplatin group (Figures 2C,D). These findings suggest that silencing circASH1L can reduce cisplatin resistance by inhibiting cell survival, decreasing cell invasion, and promoting apoptosis in ovarian cancer cells.

### 3.3 Silencing circASH1L attenuated cisplatin resistance by regulating ferroptosis in ovarian cancer cells

**FIGURE 3:**
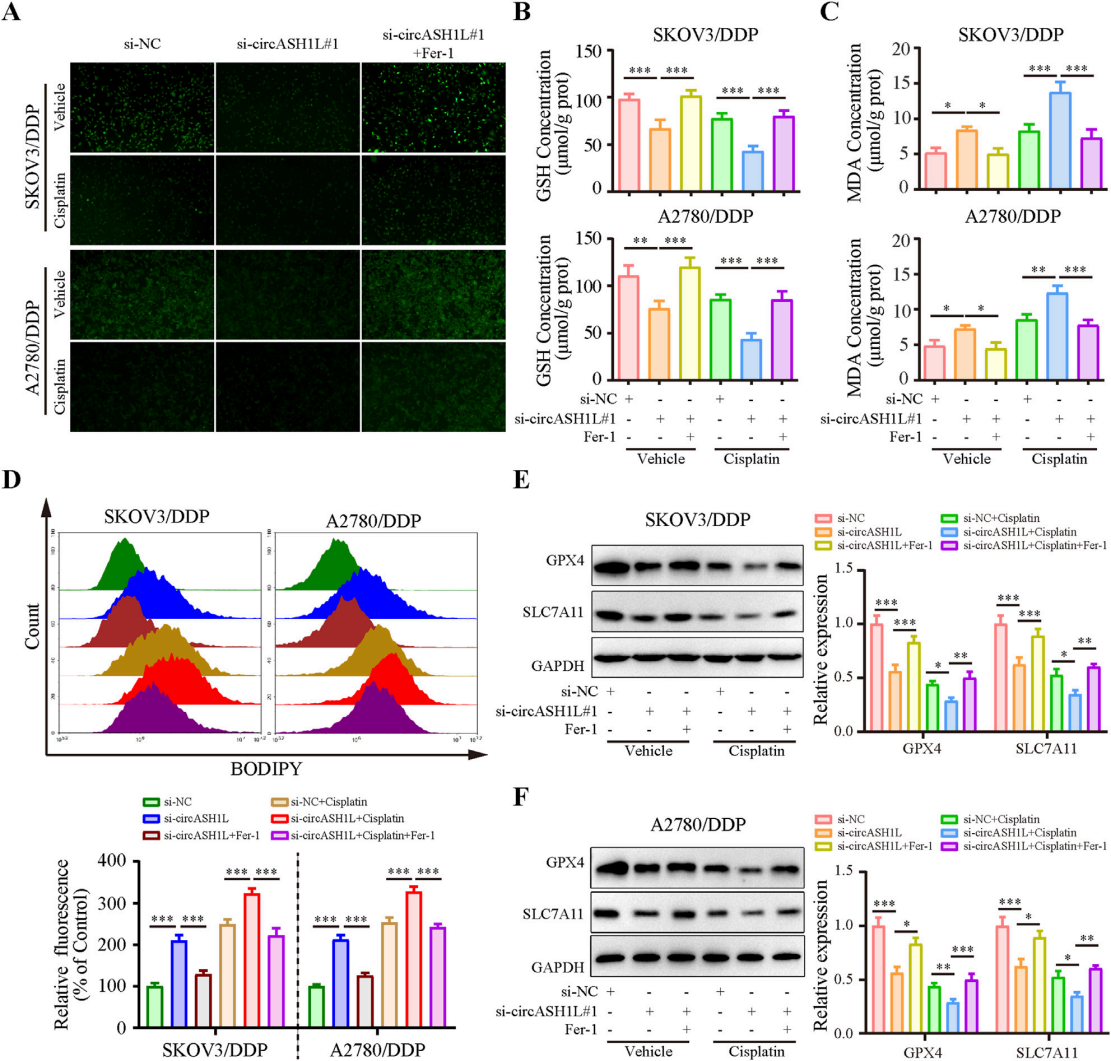
CircASH1L silencing promoted ferroptosis in cisplatin-resistant ovarian cancer cells SKOV3/DDP and A2780/DDP cells were treated with si-NC, si-circASH1L, or si-RNA + Fer-1 (ferroptosis inhibitor), with or without cisplatin. **(A)** Intracellular iron ions were detected using the PGSK probe. **(B)** Intracellular glutathione (GSH) concentration was determined using a GSH assay kit. **(C)** Malondialdehyde (MDA) levels were determined using an MDA assay kit. **(D)** Lipid peroxidation was assessed by flow cytometry with BODIPY 581/591 C11 staining. Western blot analysis showed ferroptosis markers GPX4 and SLC7A11 expression in **(E)** SKOV3/DDP and **(F)** A2780/DDP cells, with GAPDH as a loading control. Data were presented as the mean ± SD of three independent experiments. *p < 0.05. **p < 0.01, ***p < 0.001.

Given the close relationship between ferroptosis and drug resistance, we investigated the effect of circASH1L on ferroptosis. As shown in Figure 3A, the iron ions concentration was significantly increased (with weak PGSK fluorescence intensity) in the presence of si-circASH1L#1. Moreover, accumulation was further aggravated when si-circASH1L#1 was combined with cisplatin treatment, compared to cisplatin treatment alone. However, ferrostatin-1 (Fer-1), a ferroptosis inhibitor, reversed the iron ions accumulation induced by circASH1L silencing and increased PGSK fluorescence intensity. Next, we observed GSH depletion and increased MDA generation with si-circASH1L#1 transfection. Silencing circASH1L enhanced cisplatin-induced GSH depletion and MDA generation. Conversely, Fer-1 treatment restored GSH levels and decreased MDA concentrations (Figures 3B,C). Subsequently, lipid peroxidation was assessed using BODIPY 581/591 C11 staining by flow cytometry. CircASH1L knockdown also increased lipid peroxidation levels in cisplatin-exposed cells, while Fer-1 treatment alleviated lipid peroxidation (Figure 3D). GPX4 and SLC7A11 are key proteins involved in ferroptosis. Western blot analysis confirmed that silencing circASH1L reduced the expression of GPX4 and SLC7A11 in cisplatin-treated cells. However, Fer-1 treatment blocked the effect of si-circASH1L#1 and partially restored the levels of GPX4 and SLC7A11 (Figures 3E,F). Collectively, these results suggest that silencing circASH1L promotes ferroptosis and thereby alleviates cisplatin resistance in ovarian cancer cells.

### 3.4 circASH1L served as a sponge for miR-515-5p

**FIGURE 4:**
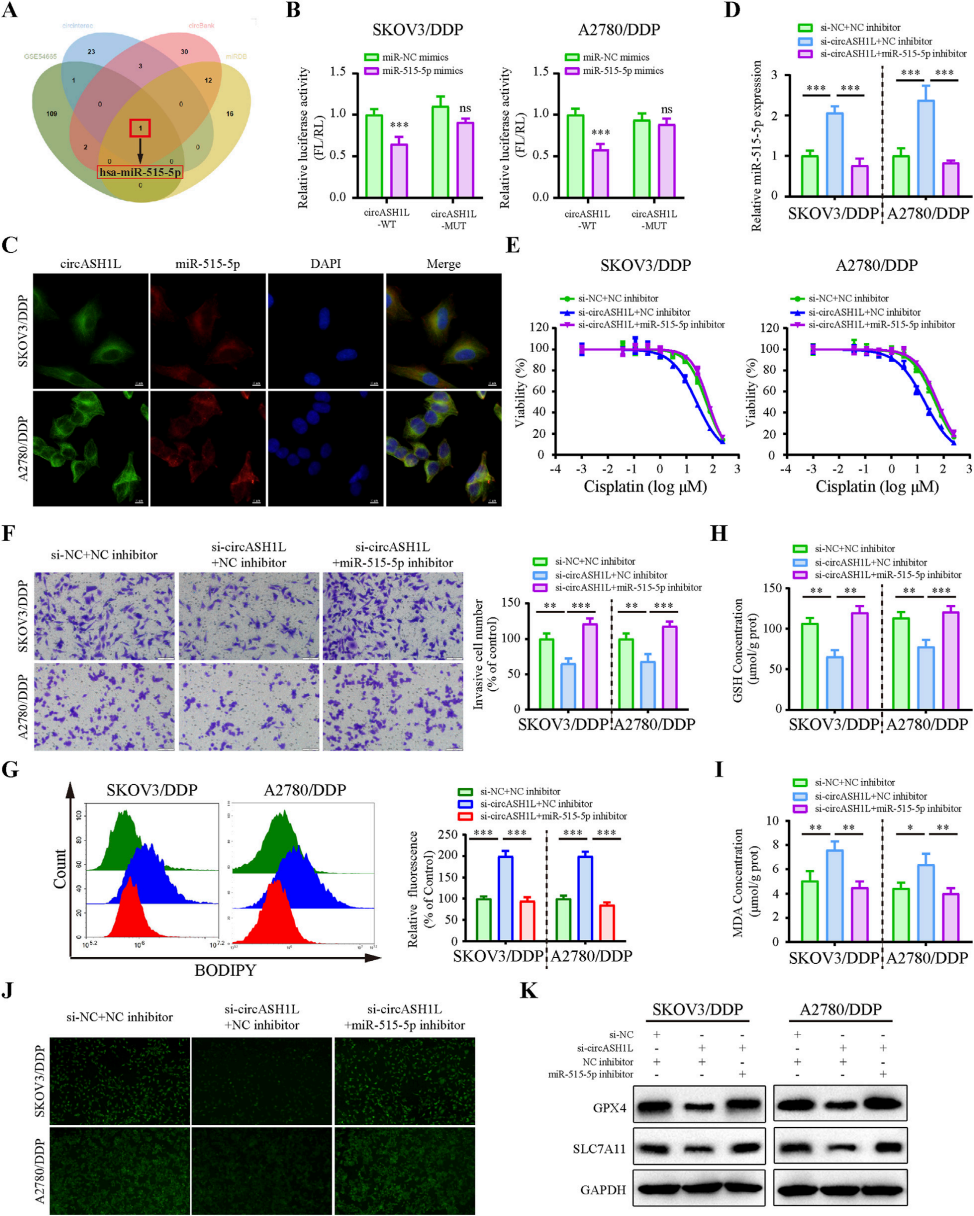
CircASH1L sponges miR-515-5p to mediate its effects on proliferation, invasion, and ferroptosis in cisplatin-resistant ovarian cancer cells **(A)** Venn diagram showed the intersection of miRNAs predicted to interact with circASH1L (from circBank, miRDB and CIRCinteractiome databases) and miRNAs significantly downregulated in cisplatin-resistant A2780 cells (from the GSE54665 dataset), identifying miR-515-3p as the only candidate miRNA. **(B)** Dual-luciferase reporter assay measured the luciferase activity in SKOV3/DDP and A2780/DDP cells co-transfected with pmirGLO-circASH1L-WT or pmirGLO-circASH1L-MUT, along with miR-515-5p mimics or negative control (NC) mimics. **(C)** Fluorescence *in situ* hybridization (FISH) analysis showed co-localization of miR-515-5p and circASH1L in the cytoplasm. The circASH1L probe was tagged with FAM, and the miR-515-3p probe was tagged with Cy3. Scale bars = 10 µm. **(D–K)** SKOV3/DDP and A2780/DDP cells were co-transfected with si-NC + NC inhibitor, si-circASH1L + NC inhibitor, or si-circASH1L + miR-515-5p inhibitor. **(D)** miR-515-5p expression levels were measured by qRT-PCR analysis. **(E)** Cell viability (% of control) was assessed by CCK-8 assay following treatment with various concentrations of cisplatin. **(F)** Transwell invasion assay was performed to evaluate cell invasion. Scale bars = 100 µm. **(G)** Lipid peroxidation was assessed by flow cytometry with BODIPY 581/591 C11 staining. **(H)** Intracellular glutathione (GSH) concentrations were determined using a GSH assay kit. **(I)** Malondialdehyde (MDA) levels were determined using an MDA assay kit. **(J)** Intracellular iron ions were detected using the PGSK probe. **(K)** Western blot analysis of ferroptosis markers GPX4 and SLC7A11 expression, with GAPDH as a loading control. Data were presented as the mean ± SD of three independent experiments. *p < 0.05. **p < 0.01, ***p < 0.001.

Considering that circASH1L is predominantly localized in the cytoplasm, we hypothesized that it may function as a miRNA sponge. To investigate whether circASH1L regulates cisplatin resistance by interacting with miRNAs, we analyzed downregulated miRNAs in A2780/DDP cells from the GEO (Gene Expression Omnibus) dataset (GSE54665) and predicted potential miRNAs that could bind to circASH1L using multiple prediction tools, including circBank, miRDB and CIRCinteractiome. By intersecting these datasets, miR-515-5p was selected as the candidate (Figure 4A). The GSE54665 dataset analysis indicated that miR-515-5p expression was downregulated in A2780/DDP cells compared to control cells (Supplementary Figure S3A). In addition, the expression of miR-515-5p in cisplatin-resistant patients was significantly lower than that in cisplatin-susceptible group (*p* < 0.05), suggesting a potential association between miR-515-5p downregulation and cisplatin resistance (Supplementary Figure S3B). Kaplan-Meier analysis further demonstrated that ovarian cancer patients with higher miR-515-5p expression exhibited significantly longer overall survival compared to those with low expression (*p* < 0.05) (Supplementary Figure S3C).

The predicted binding sites between circASH1L and miR-515-5p are shown in Supplementary Figure S3D. To confirm the binding of miR-515-5p to circASH1L, a dual-luciferase reporter assay was conducted. As shown in Figure 4B, miR-515-5p significantly decreased the luciferase activity of pmirGLO-circASH1L-WT, while it had almost no effect on pmirGLO-circASH1L-MUT luciferase activity in A2780/DDP and SKOV3/DDP cells. Co-localization of circASH1L and miR-515-5p was analyzed by FISH, revealing that both circASH1L and miR-515-5p are located in the cytoplasm (Figure 4C). These findings suggest that circASH1L directly interacts with miR-515-5p and regulates its expression.

To investigate whether circASH1L induces cisplatin resistance through miR-515-5p sponging, we silenced miR-515-5p using specific inhibitors in circASH1L-silenced A2780/DDP and SKOV3/DDP cells. As shown in Figure 4D, miR-515-5p expression was significantly elevated in circASH1L-silenced cells, and miR-515-5p inhibitors reversed this upregulation. Consistent with this, A2780/DDP and SKOV3/DDP cells were more susceptible to cisplatin treatment in the presence of si-circASH1L#1 compared to si-NC. However, miR-515-5p inhibitors reversed the effect of si-circASH1L#1, restoring cisplatin resistance (Figure 4E; Supplementary Figure S3E). Next, we examined the effect of circASH1L silencing and miR-515-5p inhibition on cell invasion. Upregulation of miR-515-5p (si-circASH1L + NC inhibitors) reduced cell invasion, whereas downregulation of miR-515-5p (si-circASH1L + miR-515-5p inhibitor) increased cell invasion (Figure 4F). To assess ferroptosis, we measured iron ions levels, lipid peroxidation, GSH, and MDA concentrations, as well as the expression of ferroptosis markers GPX4 and SLC7A11. Silencing circASH1L led to increased lipid peroxidation, iron ions, and MDA accumulation, along with decreased GSH levels and reduced GPX4 and SLC7A11 expression. In contrast, treatment with miR-515-5p inhibitors significantly reversed these effects, promoting ferroptosis in A2780/DDP and SKOV3/DDP cells (Figures 4G–K). Thus, circASH1L acts as a sponge for miR-515-5p, and silencing circASH1L promotes ferroptosis and sensitizes cells to cisplatin through upregulation of miR-515-5p.

### 3.5 CDCA7 is a target of miR-515-5p and regulated by circASH1L

**FIGURE 5:**
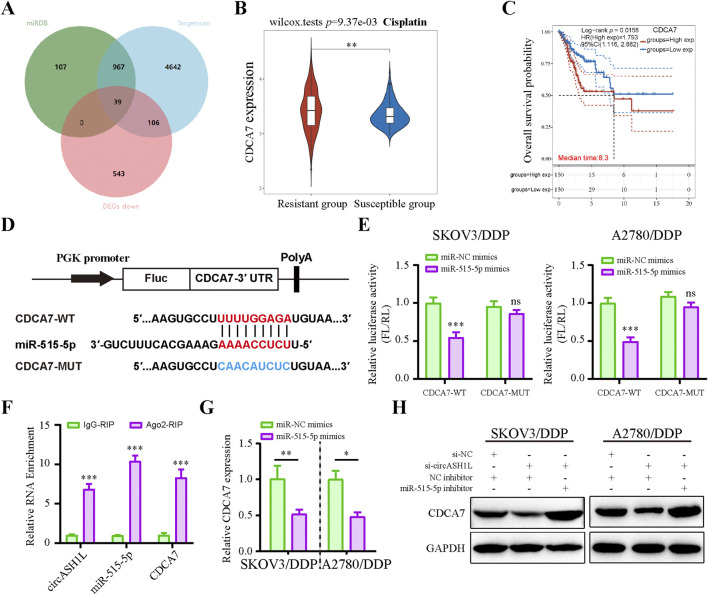
Cell cycle-related CDCA7 is a target of miR-515-5p and regulated by circASH1L **(A)** Venn diagram showed the intersection of targets predicted to interact with miR-515-5p from the Targetscan and miRDB databases, and significantly downregulated genes in erastin-treated ES2 cells from RNA-seq analysis. **(B)** Box plot analysis of CDCA7 expression in ovarian cancer patients stratified by cisplatin resistance status, based on TCGA and GEO datasets. **(C)** Kaplan–Meier survival curves depicting overall survival of ovarian cancer patients according to CDCA7 expression levels. **(D)** Diagram illustrated the design of wild-type (WT) and mutant (MUT) CDCA7 based on the predicted binding site between miR-515-5p and CDCA7. **(E)** Dual-luciferase reporter assay measured luciferase activity in SKOV3/DDP and A2780/DDP cells co-transfected with pmirGLO-CDCA7-WT or pmirGLO-CDCA7-MUT, along with miR-515-5p mimics or negative control (NC) mimics. **(F)** AGO2 RIP assay showed the enrichment of circASH1L, miR-515-5p, and CDCA7 by Ago2 antibody in A2780/DDP cells. **(G)** qRT-PCR analysis of CDCA7 expression in SKOV3/DDP and A2780/DDP cells transfected with miR-515-5p mimics or negative control (NC) mimics. **(H)** Western blot analysis of CDCA7 expression in SKOV3/DDP and A2780/DDP cells co-transfected with si-NC + NC inhibitor, si-circASH1L + NC inhibitor, or si-circASH1L + miR-515-5p inhibitor, with GAPDH as a loading control. Data were presented as the mean ± SD of three independent experiments. *p < 0.05. **p < 0.01, ***p < 0.001.

Potential miR-515-5p target genes in ovarian cancer cells were investigated using the Targetscan and miRDB databases, combined with DEGs positively associated with circASH1L expression from transcriptome data treated with erastin (Figure 5A). Among these, cell division cycle associated 7 (CDCA7) attracted our attention due to its emerging role in the cell cycle and ferroptosis regulation in cancer cells. TCGA data from the GEPIA database suggested that CDCA7 expression was significantly increased in ovarian cancer tissues compared to adjacent non-cancerous tissues (*p* < 0.05) (Supplementary Figure S4A). UALCAN analysis revealed that low CDCA7 expression was correlated with low tumor grade, while the expression of CDCA7 progressively increased from stage 2 to stage 3 in ovarian cancer samples (Supplementary Figure S4B). Moreover, as shown in Figure 5B, the expression of CDCA7 in cisplatin-resistant patients was significantly higher than that in cisplatin-susceptible group (*p* < 0.05). Kaplan-Meier analysis further demonstrated that patients exhibiting higher CDCA7 expression had poorer overall survival compared to those with lower expression levels (*p* < 0.05) (Figure 5C). Moreover, the starBase database indicated a negative correlation between miR-515-5p and CDCA7, although this correlation did not reach statistical significance, suggesting that it should be considered for further investigation (Supplementary Figure S4C). The predicted binding site between miR-515-5p and CDCA7 is shown in Figure 5D. A dual-luciferase reporter assay was performed in A2780/DDP and SKOV3/DDP cells, and the results confirmed that the 3′-UTR of CDCA7-WT was a downstream target of miR-515-5p, whereas no such interaction was observed with the CDCA7-MUT (Figure 5E). The direct interaction among circASH1L, miR-515-5p, and CDCA7 was verified by Ago2-RIP analysis, which showed that all three were enriched with the anti-Ago2 antibody compared to the control IgG (Figure 5F). Next, CDCA7 expression was assessed in NC and miR-515-5p mimics-transfected cells. CDCA7 expression decreased with miR-515-5p mimics treatment compared to the NC mimics (Figure 5G). Consistently, si-circASH1L#1 decreased CDCA7 expression, while miR-515-5p inhibitors blocked the effect of si-circASH1L#1 and enhanced CDCA7 expression at the protein level (Figure 5H). In summary, circASH1L acts as a sponge for miR-515-5p and regulates the expression of CDCA7 in ovarian cancer cells.

### 3.6 CDCA7 overexpression reversed the effect of circASH1L knockdown

**FIGURE 6:**
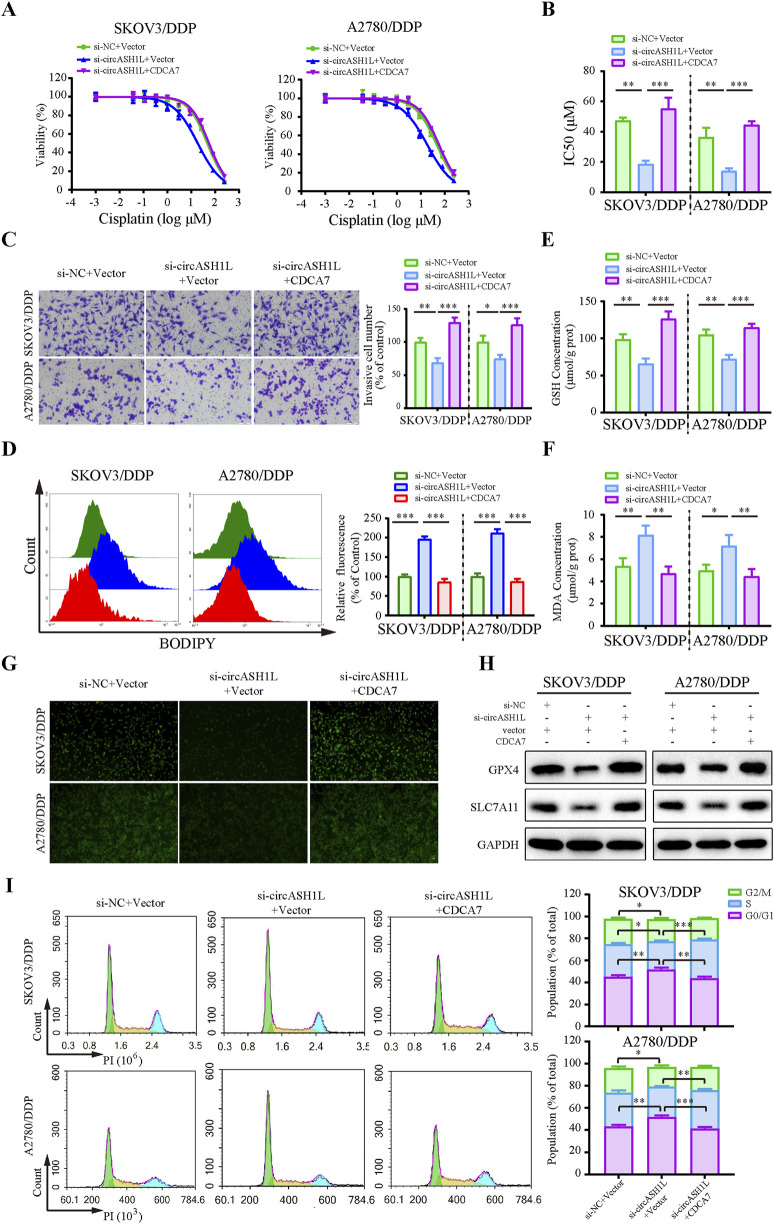
CircASH1L regulates CDCA7 to affect proliferation, invasion, ferroptosis, and cell cycle in cisplatin-resistant ovarian cancer cells SKOV3/DDP and A2780/DDP cells were co-transfected with si-NC + Vector, si-circASH1L + Vector, or si-circASH1L + CDCA7. **(A)** Cell viability (% of control) was assessed using a CCK-8 assay following treatment with various concentrations of cisplatin. **(B)** The IC50 values for cisplatin were calculated. **(C)** Transwell invasion assays were conducted to evaluate cell invasion. Scale bars = 100 µm. **(D)** Lipid peroxidation was assessed by flow cytometry with BODIPY 581/591 C11 staining. **(E)** Intracellular glutathione (GSH) concentrations were determined using a GSH assay kit. **(F)** Malondialdehyde (MDA) levels were determined using an MDA assay kit. **(G)** Intracellular iron ions were detected using the PGSK probe. **(H)** Western blot analysis of ferroptosis markers GPX4 and SLC7A11 expression, with GAPDH as a loading control. **(I)** Cell cycle was assessed by flow cytometry following propidium iodide (PI) staining. Data were presented as the mean ± SD of three independent experiments. *p < 0.05. **p < 0.01, ***p < 0.001.

To investigate the role of CDCA7 in circASH1L-mediated chemoresistance, a rescue experiment was performed by overexpressing CDCA7 in circASH1L-silenced A2780/DDP and SKOV3/DDP cells. The IC50 value for cisplatin was significantly decreased in si-circASH1L#1-transfected cells compared to the si-NC group. However, CDCA7 overexpression reversed the effect of si-circASH1L#1 and promoted chemoresistance in both A2780/DDP and SKOV3/DDP cells (Figures 6A,B). Additionally, CDCA7 overexpression enhanced cell invasion in circASH1L-silenced cells compared to the si-circASH1L#1 + vector group (Figure 6C). The accumulation of lipid peroxidation, iron ions, and MDA, as well as the reductions in GSH, GPX4, and SLC7A11 levels induced by circASH1L knockdown, were completely reversed with overexpression of CDCA7 (Figures 6D–H). Due to the close relationship between CDCA7 and cell cycle in tumor cells (Wang et al., 2019; Cai et al., 2021), we explore the impact of circASH1L-mediated regulation of CDCA7 on cell cycle progression. The results indicated that circASH1L silencing caused G0/G1 cell cycle arrest, whereas CDCA7 overexpression reversed this effect and accelerated the cell cycle in A2780/DDP and SKOV3/DDP cells (Figure 6I). In summary, these findings demonstrate that CDCA7 can abrogate the function of circASH1L in regulating ferroptosis and chemoresistance. The circASH1L/miR-515-5p/CDCA7 axis plays a critical role in these processes.

### 3.7 CDCA7 regulates ferroptosis by interacting with RRM2 and enhancing its stability

**FIGURE 7:**
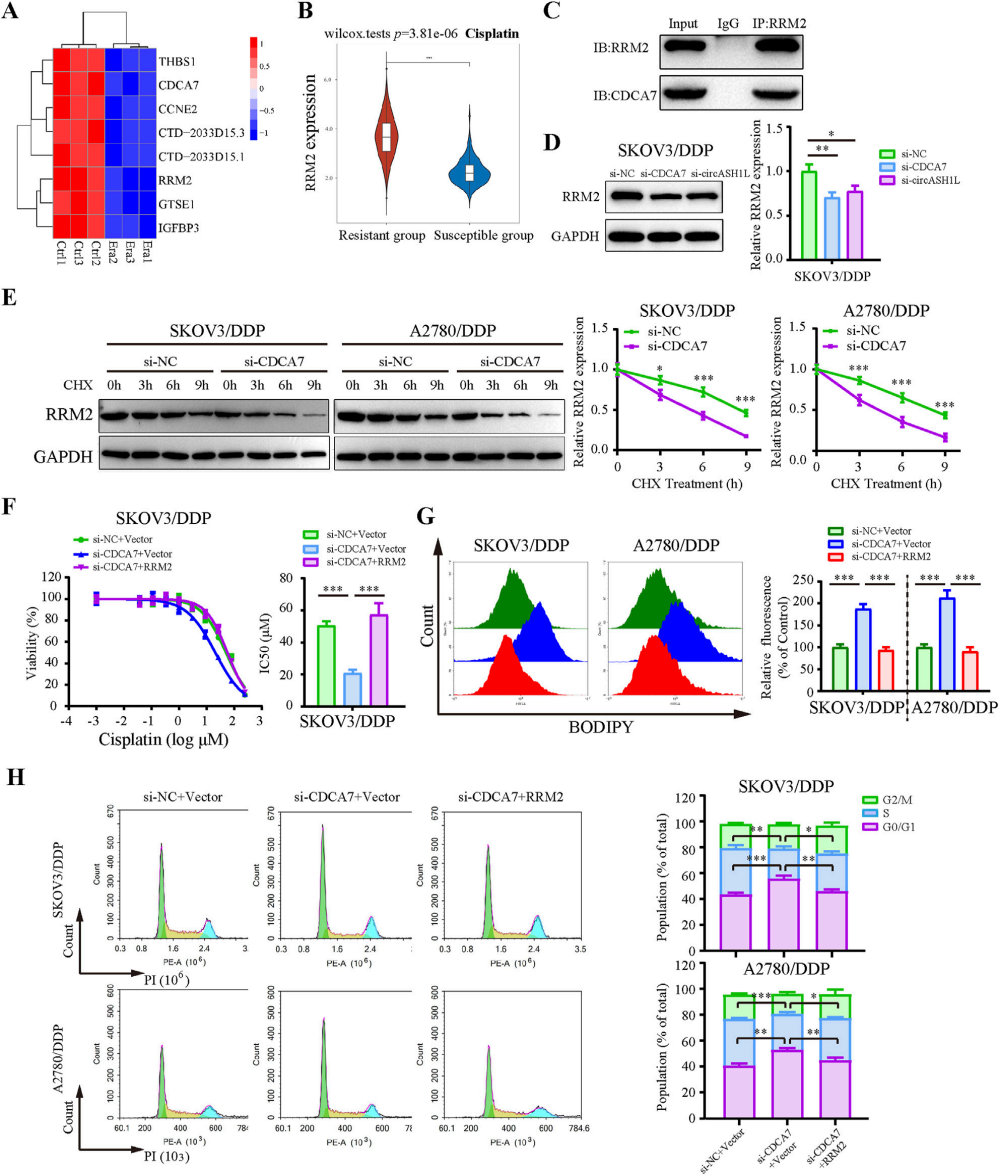
CDCA7 interacted with RRM2 and enhanced RRM2 protein stability **(A)** Heatmap showed the gene expression profile of downregulated differentially expressed genes enriched in the P53 signaling pathway in erastin-treated A2780 cells from RNA-seq. **(B)** Box plot analysis of RRM2 expression in ovarian cancer patients stratified by cisplatin resistance status, based on TCGA and GEO datasets. **(C)** Co-immunoprecipitation (Co-IP) analysis of the interaction between CDCA7 and RRM2. Lysates prepared from A2780/DDP cells were immunoprecipitated with an anti-RRM2 antibody, and the resulting complexes were analyzed by Western blot using antibodies against both RRM2 and CDCA7. **(D)** SKOV3/DDP cells were transfected with si-NC, si-CDCA7, or si-circASH1L, and Western blot analysis was performed to detect RRM2 expression, with GAPDH as a loading control. **(E)** Protein stability assay of RRM2. SKOV3/DDP and A2780/DDP cells were transfected with si-NC or si-CDCA7 and treated with 100 μg/mL cycloheximide (CHX) for 0, 3, 6, and 9 h, respectively. Western blot analysis was used to assess RRM2 protein stability. **(F)** Cell viability (% of control) was assessed by CCK-8 assay following treatment with various concentrations of cisplatin in SKOV3/DDP cells. **(G)** Lipid peroxidation was assessed by flow cytometry with BODIPY 581/591 C11 staining. **(H)** Cell cycle was assessed by flow cytometry following propidium iodide (PI) staining. Data were presented as the mean ± SD of three independent experiments. *p < 0.05. **p < 0.01, ***p < 0.001.

Previous studies have reported that CDCA7 may regulate ferroptosis through interactions with p53 signaling pathway proteins (Wang et al., 2023). To investigate this, we analyzed RNA-seq data and identified significantly downregulated p53 pathway proteins in erastin-treated A2780 cells that were positively correlated with CDCA7 expression (Figure 7A). Based on predictions from the STRING database, RRM2 (Ribonucleotide reductase regulatory subunit M2), a member of the downregulated proteins in the p53 signaling pathway, was identified as a candidate to interact with CDCA7 (Supplementary Figure S5A). The starBase database analysis showed a positive correlation between CDCA7 and RRM2 expression in ovarian cancer (*p* < 0.01) (Supplementary Figure S5B). Moreover, the GEPIA database analysis indicated that RRM2 expression was significantly higher in ovarian cancer tissues compared to adjacent non-cancerous tissues (*p* < 0.05) (Supplementary Figure S5C). Clinical sample analysis further demonstrated that the expression of RRM2 in cisplatin-resistant group was significantly higher than that in cisplatin-susceptible group (*p* < 0.05) (Figure 7B). Moreover, Kaplan–Meier survival analysis also demonstrated that patients with high RRM2 expression exhibited poorer overall survival compared to those with low expression (*p* < 0.05) (Supplementary Figure S5D). The interaction between CDCA7 and RRM2 was confirmed by Co-IP assay (Figure 7C). Additionally, the expression of RRM2 was downregulated by si-circASH1L#1 or si-CDCA7 (Figure 7D; Supplementary Figure S5E), suggesting that CDCA7 may regulate RRM2 protein expression. To evaluate whether CDCA7 influences the protein stability of RRM2, A2780/DDP and SKOV3/DDP cells were treated with cycloheximide (CHX), a protein synthesis inhibitor, after transfection with si-NC or si-CDCA7. As expected, the half-life of RRM2 was shortened by CDCA7 silencing compared to si-NC transfection (Figure 7E), indicating that CDCA7 stabilizes RRM2 protein.

Rescue experiments further showed that overexpression of RRM2 in CDCA7-silenced cells partially reversed the effects of CDCA7 knockdown, including increased cisplatin sensitivity and ferroptosis-related changes such as elevated lipid peroxidation, intracellular iron accumulation, and MDA levels, as well as GSH content and G0/G1 cell cycle arrest (Figures 7F–H; Supplementary Figure S5). Collectively, these findings suggest that CDCA7 regulates ferroptosis and chemoresistance at least in part by stabilizing RRM2.

### 3.8 Silencing circASH1L promoted ferroptosis and increased cisplatin sensitivity *in vivo*

**FIGURE 8:**
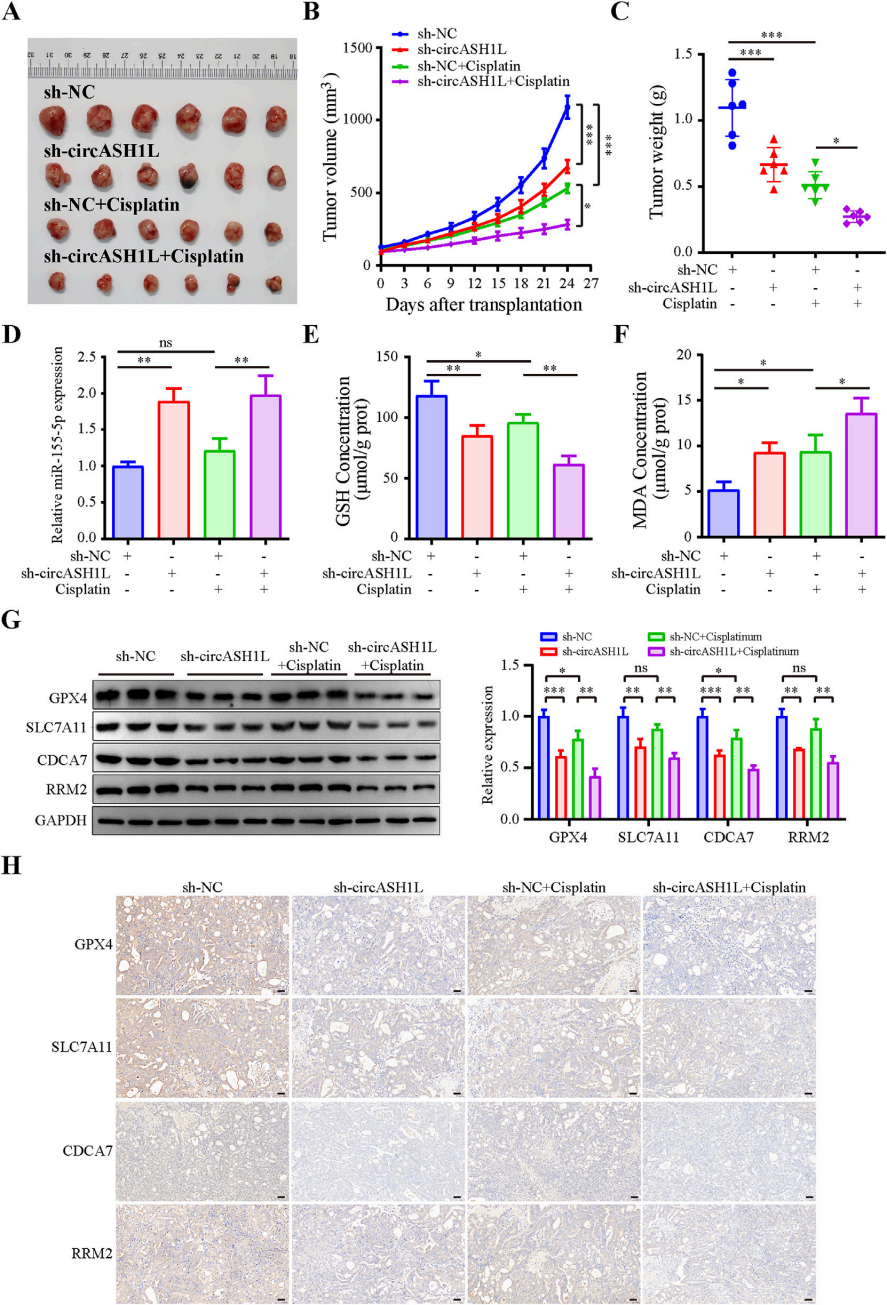
CircASH1L regulated the miR-515-5p/CDCA7/RRM2 axis to induce ferroptosis-mediated tumor inhibition and cisplatin resistance in the A2780/DDP xenograft mouse model. A2780/DDP cells stably transfected with either sh-NC or sh-circASH1L were subcutaneously inoculated into Balb/C-nude mice (5 × 10^6^ cells per mouse). The mice were treated with either PBS or cisplatin (6 mg/kg) twice a week for a total of five doses (n = 6 per group). Body weights and tumor volumes were monitored every three days. **(A)** Images of tumor tissues. **(B)** Tumor volume curve. **(C)** Body weight curve. **(D)** qRT-PCR analysis of miR-515-5p expression in tumor tissues. **(E)** Glutathione (GSH) levels and **(F)** malondialdehyde (MDA) levels in tumor tissues were measured using GSH and MDA assay kits. **(G)** Western blot analysis of GPX4, SLC7A11, CDCA7, and RRM2 expression levels in tumor tissue samples, with GAPDH as a loading control. **(H)** IHC staining to detect the expression of GPX4, SLC7A11, CDCA7, and RRM2 in tumor tissues. Scale bars = 50 μm. Data were presented as the mean ± SD of three independent experiments. *p < 0.05. **p < 0.01, ***p < 0.001.

To investigate the effect of circASH1L on cisplatin treatment *in vivo*, a xenograft mouse model was established using A2780/DDP cells stably transfected with either sh-NC or sh-circASH1L. The mice were treated with either vehicle (PBS) or cisplatin. As shown in Figures 8A–C and Supplementary Figure S6A, silencing circASH1L or cisplatin treatment significantly suppressed tumor volume and weight. Additionally, silencing circASH1L enhanced the inhibitory effect of cisplatin on tumor growth (cisplatin + sh-NC vs. cisplatin + sh-circASH1L, *p* < 0.05). qRT-PCR analysis revealed that circASH1L expression was markedly downregulated in the vehicle + sh-circASH1L or cisplatin + sh-circASH1L groups (Supplementary Figure S6B). In contrast, miR-515-5p expression was significantly upregulated in these groups (Figure 8D). Furthermore, silencing circASH1L significantly induced ferroptosis in the tumor tissues, as evidenced by the accumulation of MDA and the depletion of GSH (Figures 8E,F). Western blot analysis showed that sh-circASH1L accelerated the cisplatin-induced reduction of GPX4 and SLC7A11 expression, as well as the downregulation of CDCA7 and RRM2 in tumor tissues (Figure 8G). The same trends were observed by IHC analysis (Figure 8H). In summary, silencing circASH1L promotes ferroptosis, enhances chemosensitivity to cisplatin, and inhibits the progression of ovarian cancer cells *in vivo*.

## 4 Discussion

Ovarian cancer is one of the most common and complex gynecological malignancies. Over the past few decades, platinum-based treatments, particularly cisplatin, have been the mainstay of therapy for ovarian cancer (Ranasinghe et al., 2022). However, chemoresistance remains a significant challenge for most patients, leading to tumor recurrence and poor prognosis (Kapper et al., 2024). Recent studies have identified key mechanisms underlying chemoresistance, including reduced cisplatin accumulation (Shen et al., 2012), enhanced DNA repair (Basu and Krishnamurthy, 2010), and evasion of apoptosis (Ghosh, 2019). Emerging evidence suggests that combining ferroptosis inducers with chemotherapy results in remarkable synergistic effects in cancer treatment (Wang X. et al., 2024). Therefore, exploring ferroptosis as a therapeutic strategy may help overcome drug resistance, providing new insights into the treatment of ovarian cancer and improving patient survival.

circRNAs belong to the noncoding RNA family and play crucial roles in regulating chemoresistance in various cancers (Xin et al., 2021). Given the emerging importance of ferroptosis in cancer suppression, we sought to explore circRNAs involved in ferroptosis and elucidate their role in overcoming cisplatin resistance in ovarian cancer. Our RNA-seq results identified three circRNAs that were significantly downregulated after treatment with the ferroptosis inducer erastin, drawing our attention. Further investigation revealed that circASH1L was notably upregulated in cisplatin-resistant A2780/DDP and SKOV3/DDP cells. Previous studies have shown that circASH1L can attenuate ferroptosis and promote skin wound healing (Zha et al., 2023; Wang Q. et al., 2024). However, the effect of circASH1L on ferroptosis and drug resistance in ovarian cancer had not been previously explored.

In the present study, we found that silencing circASH1L alleviates cisplatin resistance in A2780/DDP and SKOV3/DDP cells, as demonstrated by reduced cell viability, decreased invasion, and increased apoptosis. Further analysis showed that circASH1L knockdown induces iron accumulation, MDA generation, GSH depletion, and lipid peroxidation, all hallmark features of ferroptosis (Wang X. et al., 2024). GPX4 has been identified as a critical factor in ferroptosis induced by RSL3 and erastin, with its inhibition leading to lipid peroxidation and subsequent ferroptosis (Yang et al., 2014). SLC7A11, a functional subunit of the system Xc-responsible for GSH synthesis, is suppressed by p53, promoting ferroptosis (Jiang et al., 2015). Similarly, our study shows that silencing circASH1L inhibits the expression of GPX4 and SLC7A11, key regulators of ferroptosis. It is well established that cisplatin exerts its anti-cancer effects primarily by generating DNA damage in the nucleus, which leads to the induction of apoptosis (Zamble and Lippard, 1995). Interestingly, recent studies suggest that cisplatin can also enhance ferroptosis by inducing GSH depletion and inhibiting GPX4, providing a novel mechanism for cancer therapy (Guo et al., 2018). Additionally, cisplatin-induced oxidative stress and lipid peroxide formation make cancer cells more susceptible to ferroptosis (Kapper et al., 2024). This may explain the synergistic effect observed when silencing circASH1L in combination with cisplatin treatment, as well as the use of ferroptosis inducers in combination with cisplatin in other studies (Chen et al., 2023; Qin et al., 2023).

Numerous studies have demonstrated that circRNAs exert their functions through mechanisms such as sponging miRNAs, interacting with proteins, and epigenetic modifications (Najafi, 2022; 2023). Most circRNAs, derived from exonic regions of genes, are localized in the cytoplasm, where they primarily act as miRNA “sponges” (Li et al., 2022). By sequestering miRNAs, circRNAs can upregulate the expression of downstream target genes, often promoting malignant behaviors in tumor cells. For example, circSnx12 contributes to cisplatin resistance by inhibiting ferroptosis through sponging the miR-194-5p/SLC7A11 axis in ovarian cancer (Qin et al., 2023). circ0101675 promotes cell proliferation, angiogenesis, migration, and immune evasion by targeting the miR-607/PDL1 pathway in non-small cell lung cancer cells (Lu et al., 2024). In our study, bioinformatic analysis revealed that miR-515-5p is a potential target of circASH1L, which was confirmed by a dual-luciferase reporter assay. Downregulation of miR-515-5p using its inhibitors reversed the effects of circASH1L silencing on ferroptosis and chemosensitivity. miR-515-5p has been shown to play a protective role against the malignant behaviors of several cancer types, including non-small cell lung cancer (Zhao et al., 2023) and colorectal cancer (Xu et al., 2024). Our research further confirms its therapeutic effect in ovarian cancer.

The relationship between the cell cycle and ferroptosis has gained increasing attention in recent years. It is well-established that cell cycle progression, especially during the G0/G1 phase, plays a pivotal role in regulating ferroptosis. The ability of cancer cells to escape from cell cycle arrest allows them to evade ferroptosis and continue to proliferate, which contributes to chemoresistance. Previous studies have suggested that cell cycle arrest can sensitize cells to ferroptosis in a variety of cancers, including lung adenocarcinoma (Liang et al., 2024), renal cell carcinoma (Markowitsch et al., 2020), and fibrosarcoma (Rodencal et al., 2024). In this study, we found that CDCA7 is directly regulated by miR-515-5p. Functional rescue assays demonstrated that overexpression of CDCA7 reversed the suppressive effects of circASH1L silencing on cell proliferation, invasion, ferroptosis, and cell cycle progression, thereby enhancing chemoresistance in A2780/DDP and SKOV3/DDP cells. Moreover, we found that silencing circASH1L leads to G0/G1 phase arrest and promotes ferroptosis, whereas CDCA7 overexpression accelerates the cell cycle and suppresses ferroptosis. Our results indicate that circASH1L modulates ferroptosis by influencing the cell cycle and contributes to cisplatin resistance. CDCA7 is closely associated with cell cycle regulation in tumor cells. Increased CDCA7 expression is observed in lung adenocarcinoma compared to adjacent normal tissues and enhances cell proliferation via accelerating cell cycle (Wang et al., 2019). While CDCA7 knockdown arrests cell cycle and inhibits cell proliferation in ovarian cancer (Cai et al., 2021). Moreover, CDCA7 is highly expressed in glioma and has been shown to suppress ferroptosis by interacting with proteins involved in the cell cycle and p53 signaling (Wang et al., 2023).

Further studies indicated that CDCA7 interacts with RRM2 and inhibits its degradation. RRM2 is a key ferroptosis regulator, and RRM2 inhibition has been associated with the induction of ferroptosis and tumor immune infiltration (Tang et al., 2021). Interestingly, inhibiting RRM2 in hepatocellular carcinoma cells using celastrol has been shown to induce ferroptosis and suppress cell proliferation, migration, and invasion (Zhang et al., 2024). RRM2 has also been reported to be closely related to the cell cycle (Sultana et al., 2024; Yang et al., 2024b), further suggesting the critical role of cell cycle regulation in circASH1L-mediated ferroptosis. Protein-protein interactions are crucial for stabilizing proteins involved in biological processes. For instance, NPRA (Natriuretic peptide receptor A) interacts with HIF-1α, preventing its proteolysis and increasing VEGF expression to enhance angiogenesis in gastric cancer (Li et al., 2021). Similarly, NPR1 interacts with PARL, stabilizing it and contributing to cisplatin resistance in gastric cancer cells (Wu et al., 2024). Consistent with these findings, the rescue experiments showed that overexpression of RRM2 in CDCA7-silenced cells effectively reversed the ferroptosis-promoting effects, highlighting the functional significance of the CDCA7–RRM2 interaction in maintaining RRM2 stability and suppressing ferroptosis in ovarian cancer cells.

To date, most ferroptosis-related circRNAs exert their effects by targeting classical ferroptosis effectors such as GPX4 and SLC7A11 via circRNA–miRNA–mRNA regulatory axes. For instance, circBLNK suppresses ferroptosis by competitively binding miR-188-3p to upregulate GPX4 expression (Li et al., 2023), while circACAP2 similarly enhances GPX4 levels through miR-193a-5p sequestration (Liu et al., 2022). Parallel mechanisms involving SLC7A11 regulation have been identified, with circ-CDK8 modulating the miR-615-5p/SLC7A11 axis (Sun et al., 2024) and circPVT1 maintaining SLC7A11 expression via miR-143-3p sponging (Wang et al., 2022). In contrast, circASH1L may regulate ferroptosis through the miR-515-5p/CDCA7/RRM2 axis, potentially representing an alternative regulatory pathway distinct from the GPX4-or SLC7A11-centered mechanisms. This regulatory axis potentially links ferroptosis resistance with cell cycle progression, as both CDCA7 and RRM2 are important regulators of the cell cycle. These findings suggest that circASH1L could represent a distinct mechanism among ferroptosis-associated circRNAs, mediating ferroptosis resistance via cell cycle–related molecular interactions.

## 5 Conclusion

This study uncovers a novel role for circASH1L in regulating ferroptosis and chemotherapy resistance in ovarian cancer. We found that circASH1L is significantly upregulated in cisplatin-resistant ovarian cancer cells, and silencing circASH1L effectively reverses resistance and promotes ferroptosis. Mechanistically, circASH1L acts as a sponge for miR-515-5p, modulating the expression of CDCA7, which inhibits ferroptosis and enhances chemoresistance by accelerating cell cycle progression. Additionally, circASH1L may stabilize the chemoresistant phenotype through interaction with RRM2. These findings provide new insights into the role of circRNAs in overcoming chemotherapy resistance and suggest that circASH1L could serve as a potential therapeutic target to improve treatment outcomes in ovarian cancer.

## Data Availability

The original contributions presented in the study are included in the article/Supplementary Material, further inquiries can be directed to the corresponding authors.
