# Supplementary figures and images for: CircASH1L inhibits ferroptosis and enhances cisplatin resistance by sponging miR-515-5p to regulate cell cycle-related CDCA7/RRM2 in ovarian cancer cells
Source: Front Pharmacol. 2025 Jun 24;16:1563869. doi: 10.3389/fphar.2025.1563869 (PMC12235606; doi:10.3389/fphar.2025.1563869)

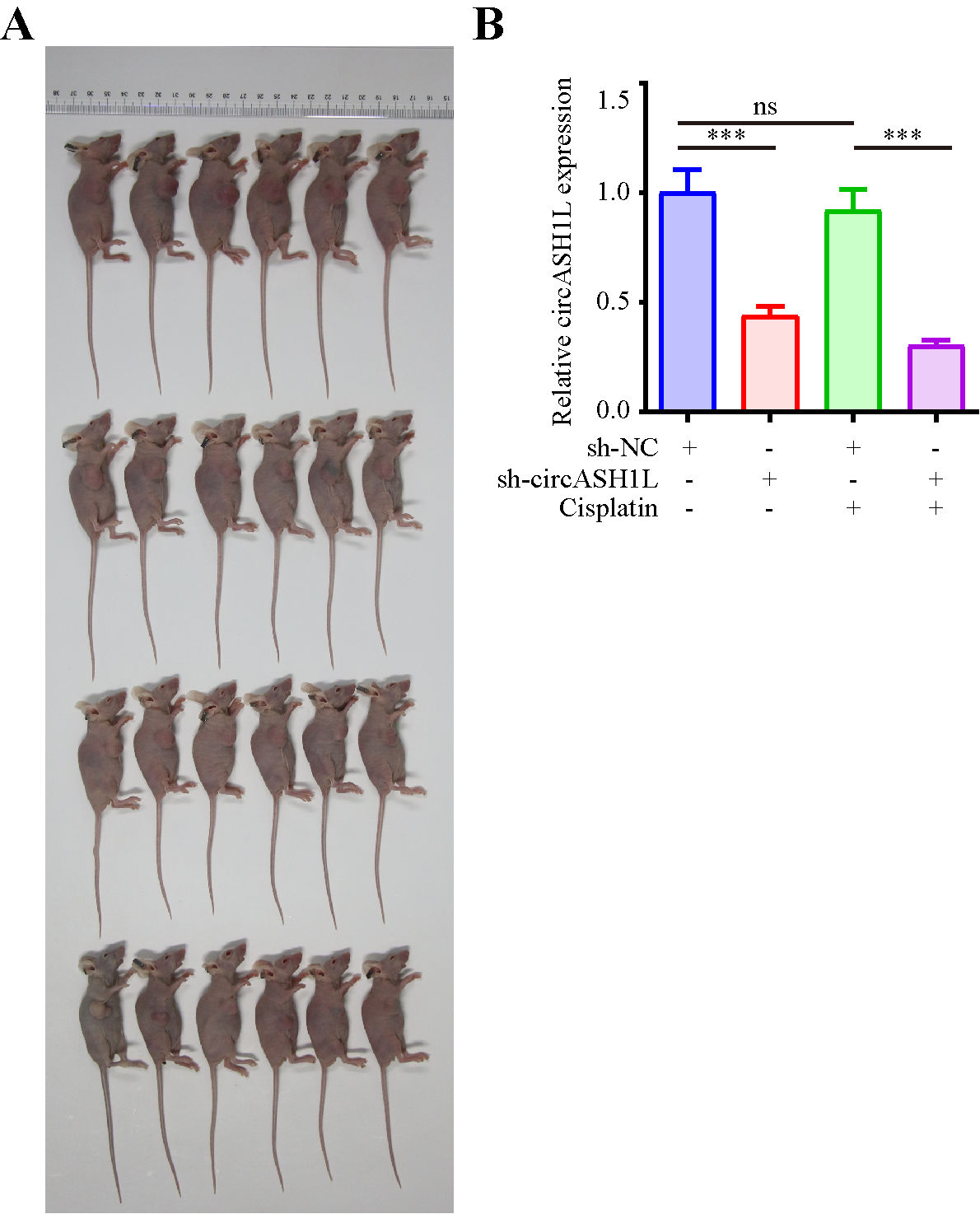

Supplement: Supplementary file 1 [file Image6.tif]

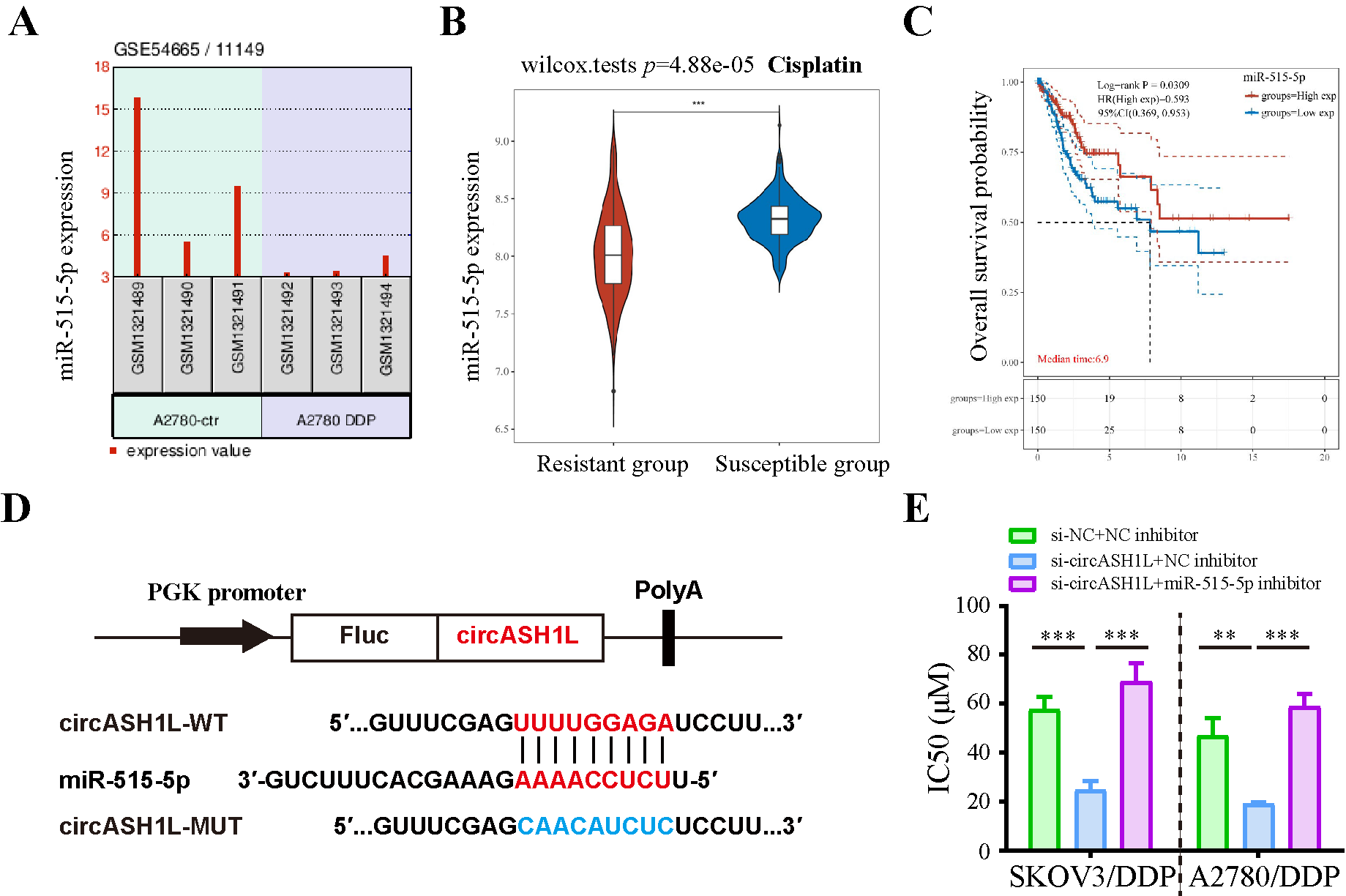

Supplement: Supplementary file 2 [file Image3.tif]

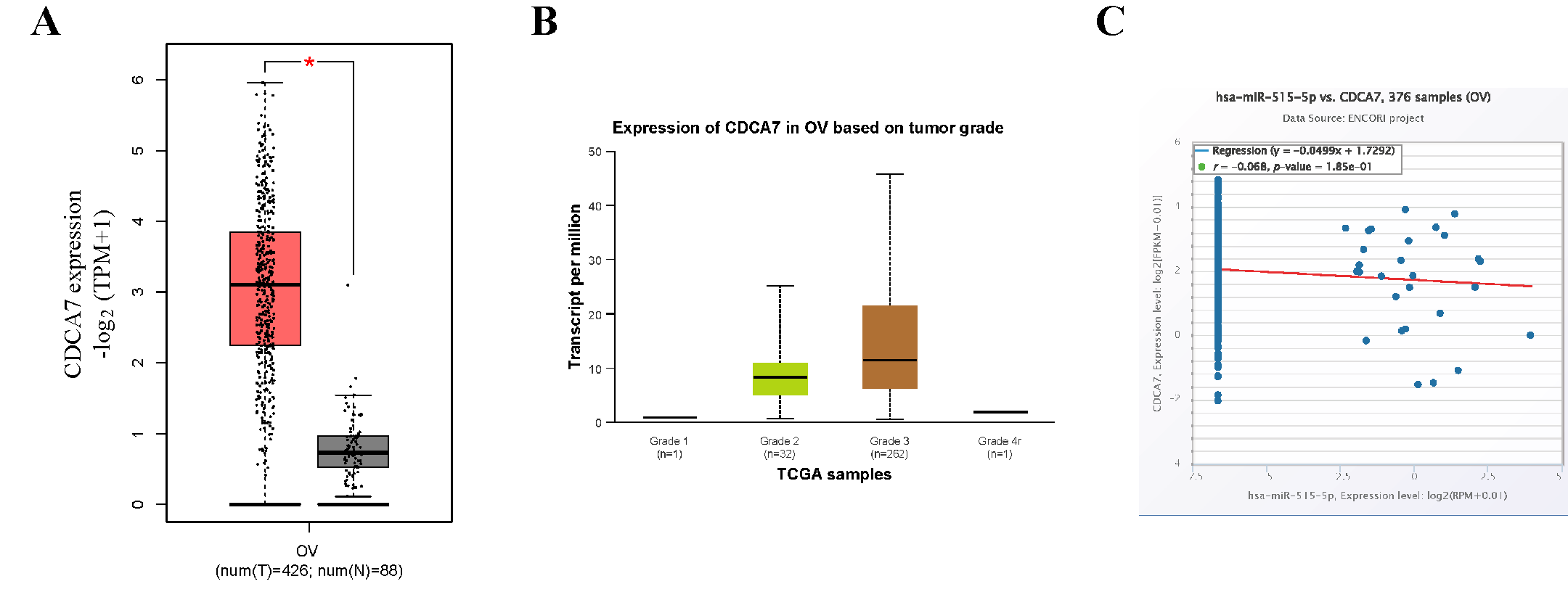

Supplement: Supplementary file 3 [file Image4.tif]

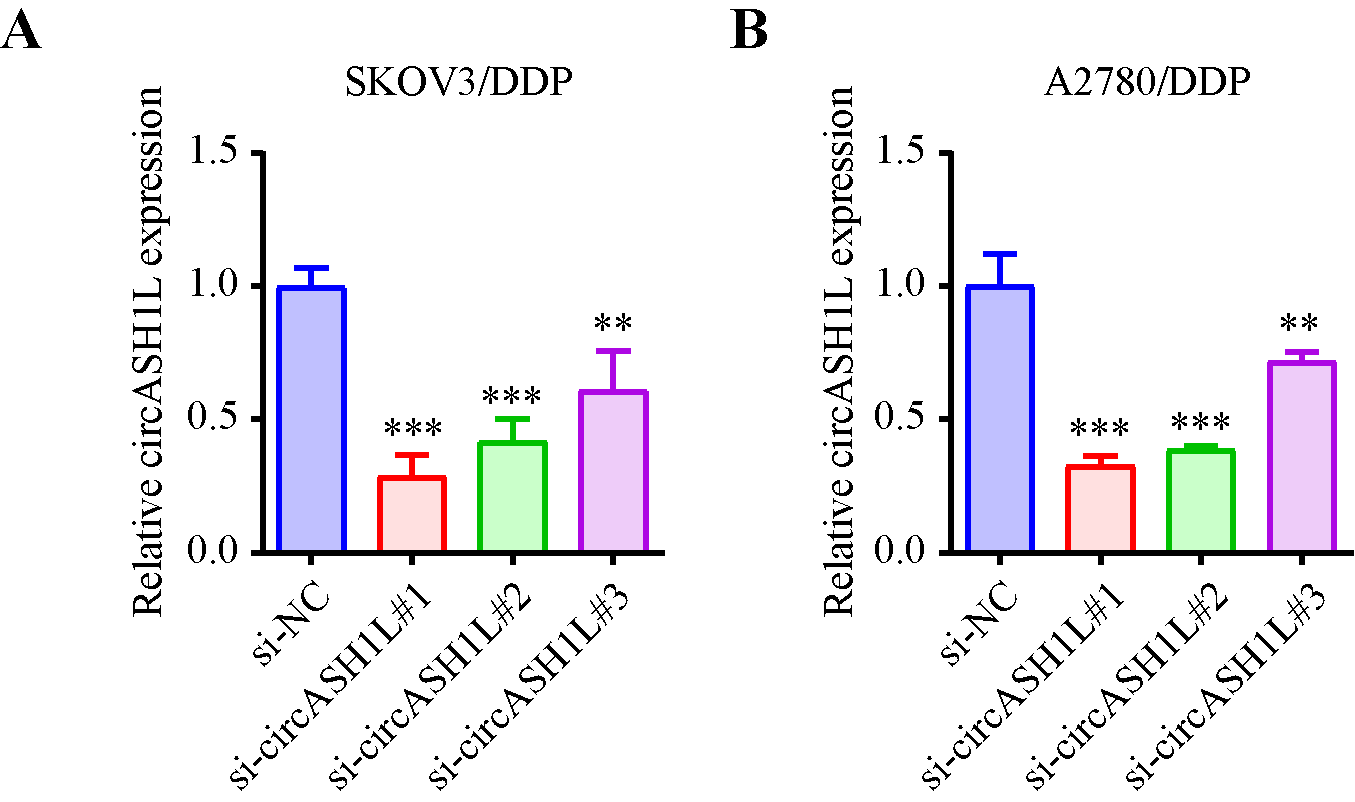

Supplement: Supplementary file 4 [file Image2.tif]

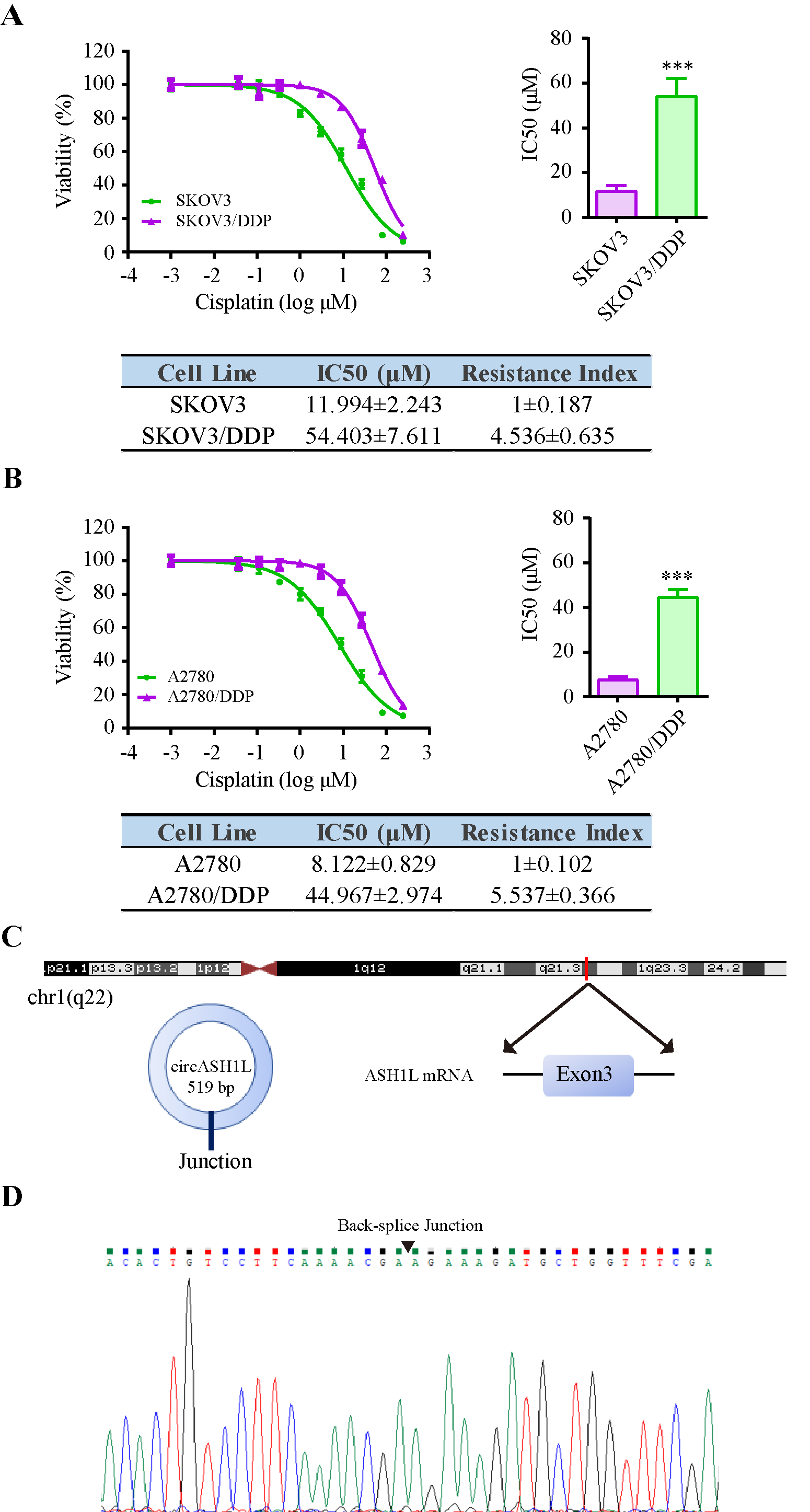

Supplement: Supplementary file 5 [file Image1.tif]

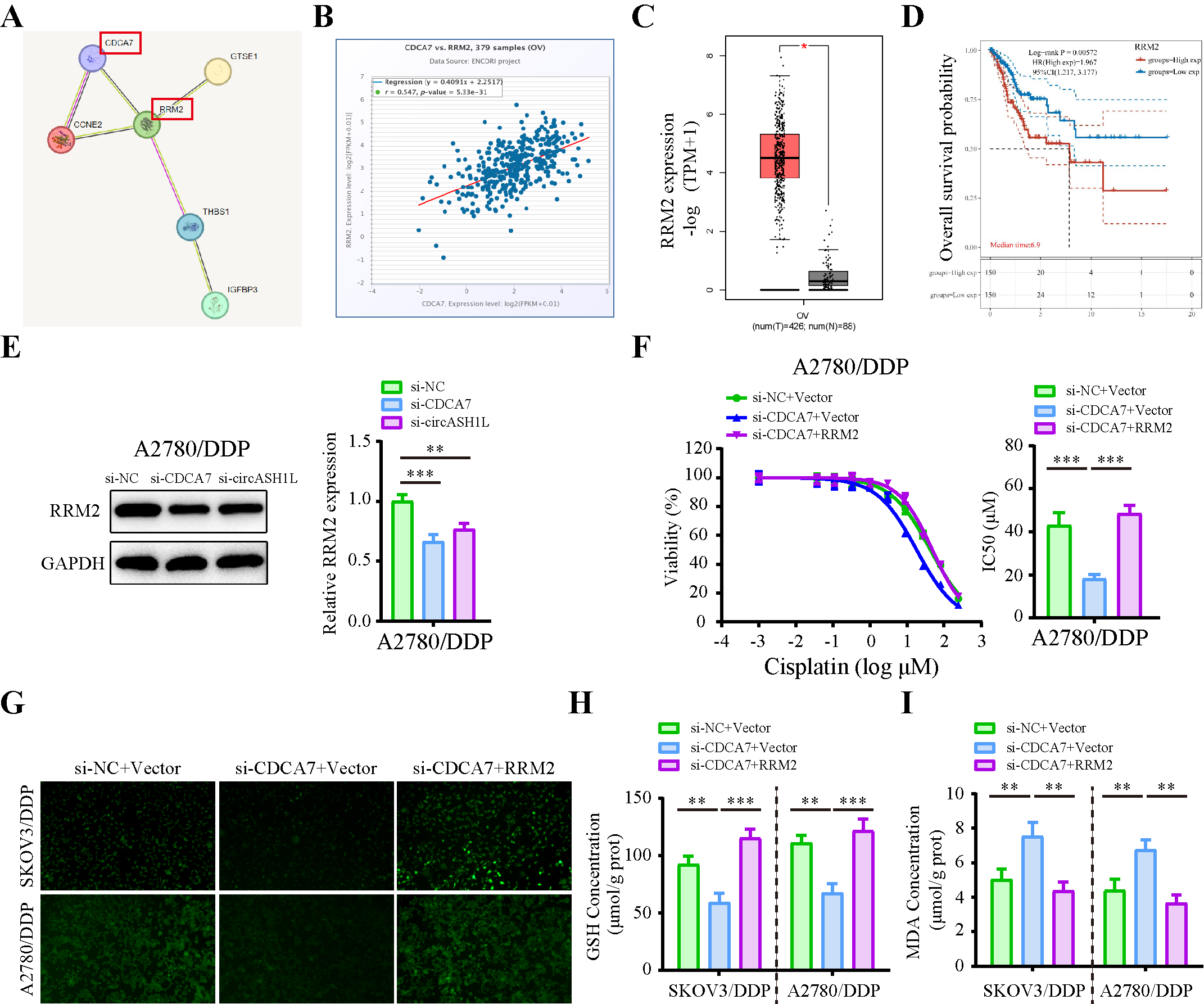

Supplement: Supplementary file 7 [file Image5.tif]
